# Mechanism and evolution of the Zn-fingernail required for interaction of VARP with VPS29

**DOI:** 10.1038/s41467-020-18773-2

**Published:** 2020-10-06

**Authors:** Harriet Crawley-Snowdon, Ji-Chun Yang, Nathan R. Zaccai, Luther J. Davis, Lena Wartosch, Emily K. Herman, Nicholas A. Bright, James S. Swarbrick, Brett M. Collins, Lauren P. Jackson, Matthew N. J. Seaman, J. Paul Luzio, Joel B. Dacks, David Neuhaus, David J. Owen

**Affiliations:** 1 MRC Laboratory of Molecular Biology Cambridge Biomedical Campus, Francis Crick Ave, Cambridge, CB2 0QH UK; 2 CIMR, The Keith Peters Building, Hills Road, Cambridge, CB2 0QQ UK; 3 Division of Infectious Disease, Department of Medicine, University of Alberta, Edmonton, Canada T6G 2G3; 4 Pharmacology and Therapeutics, The University of Melbourne, Parkville Victoria, 3010 Australia; 5 The University of Queensland, Institute for Molecular Bioscience, St Lucia, QLD 4072 Australia; 6 Department of Biological Sciences, Vanderbilt University, Nashville, TN 37232 USA

**Keywords:** Biochemistry, Endosomes, Solution-state NMR

## Abstract

VARP and TBC1D5 are accessory/regulatory proteins of retromer-mediated retrograde trafficking from endosomes. Using an NMR/X-ray approach, we determined the structure of the complex between retromer subunit VPS29 and a 12 residue, four-cysteine/Zn^++^ microdomain, which we term a Zn-fingernail, two of which are present in VARP. Mutations that abolish VPS29:VARP binding inhibit trafficking from endosomes to the cell surface. We show that VARP and TBC1D5 bind the same site on VPS29 and can compete for binding VPS29 in vivo. The relative disposition of VPS29s in hetero-hexameric, membrane-attached, retromer arches indicates that VARP will prefer binding to assembled retromer coats through simultaneous binding of two VPS29s. The TBC1D5:VPS29 interaction is over one billion years old but the Zn-fingernail appears only in VARP homologues in the lineage directly giving rise to animals at which point the retromer/VARP/TBC1D5 regulatory network became fully established.

## Abstract (web summary)

VARP is bound to endosomes and functions as a protein:protein interaction platform. Here, the authors present the NMR structure of the complex between the retromer subunit VPS29 and a VARP Zn-fingernail microdomain that is structurally distinct from Zn-fingers and further show that mutations, which abolish VPS29:VARP binding, inhibit trafficking from endosomes to the cell surface.

## Introduction

Retromer complex-based coated vesicular-tubular structures mediate retrograde transport of a vast array of transmembrane proteins of widely differing function from endosomes to the cell surface or the TGN (reviewed in refs. ^1–4^). At the heart of all retromer-based coats is a 1:1:1 core heterotrimer comprised of VPS35 (helical solenoid), VPS26 (arrestin-like fold) and VPS29 (inactive phosphatase fold), which can be identified across nearly all eukaryotic organisms^5^. Loss of retromer in mice is embryonically lethal^6,7^, and mutations in the core retromer components are linked to Alzheimer’s and Parkinson’s diseases (reviewed in ref. ^8^). In mammalian cells, retromer-based coats also contain other components including the PI3P/PI3,5P2-binding PX domain-containing sorting nexin (SNX) proteins (including SNX3, SNX-BAR proteins, and SNX27)^9–12^ and associate with regulatory proteins/protein complexes including WASH complex^13,14^, TBC1D5^15,16^, RME8^17^, EHD1 and Rabankyrin5^14,18^.

**Fig. 1:**
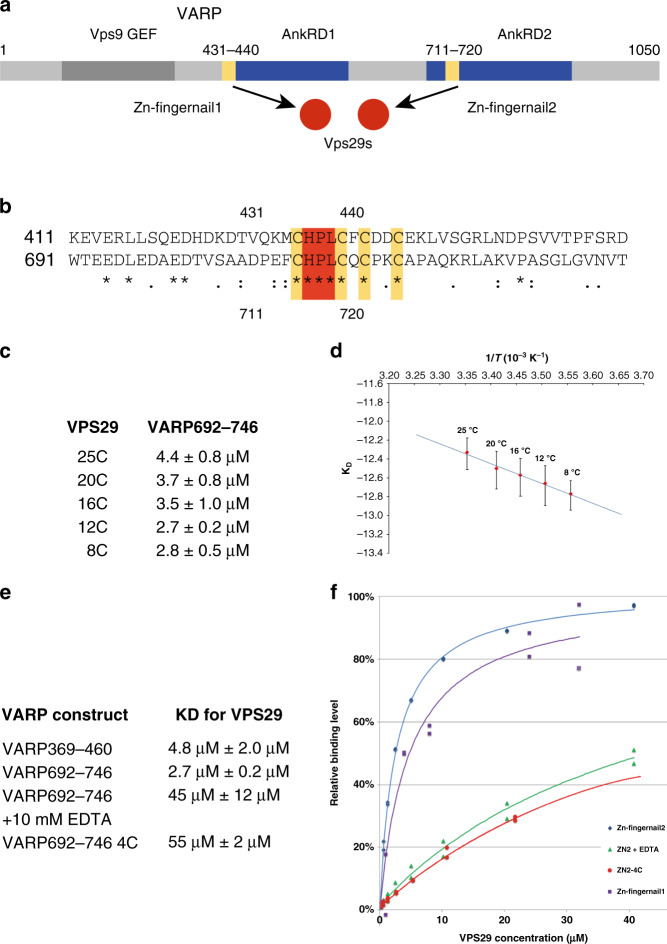
The VARP:VPS29 interaction. **a** Schematic representation of VARP with conserved cysteine motifs highlighted in yellow – designated Zn fingernails (see later). **b** Relevant sections of VARP with the conserved 4xCys motifs(yellow) and the His, Pro, Leu triplet motifs (red) highlighted. Residues identical between the two sequence sections are marked *. **c** K_D_s between short VPS29 and VARP residues 692–746 measured by SPR at five temperatures. **d** Van’t Hoff plot resulting from **c** of the interaction to estimate changes in binding enthalpy and entropy: the negative slope (Δ*H* < 0) demonstrates that the interaction is exothermic displaying favourable enthalpy and entropy changes (Δ*H*° ~ −20 kJ mol^−1^ and Δ*S*° ~ +36 J K^−1^ mol^−1^). **e**, **f** Equilibrium analyses by SPR (**e**), and resulting K_D_s (**f**), of short VPS29 binding to VARP immobilized on the sensor surface. In the absence of Zn+ + either through mutation of cysteines or treatment with EDTA binding is reduced ~20 fold.

We and others^19,20^ identified the 100 kDa, multi-domain, multifunctional endosomal protein VARP as a further regulatory/accessory factor for retromer-based coats. VARP variously binds and regulates the membrane-fusion activity of the SNARE (soluble N-ethylmaleimide attachment protein receptor) VAMP7^21,22^; is a Rab32/38 effector^19,23^; and is a GEF for Rab21/5^24^ (Fig. 1a). Other potential VARP binding partners have been reported^25^. We proposed that VARP interacts with VPS29 through a pair of duplicated ten-residue CHPLCxCxxC sequences (Fig. 1a, b), predicted on the basis of micro-PIXE analysis to be Zn^++^-binding motifs^19^ and that this interaction is responsible for stably recruiting VARP onto endosomal membranes.

Here we probe the structure and in vivo function of the complexes between these cysteine-rich, short sequences and VPS29 and analyse their mechanism and evolution in the context of other known VPS29 binding partners. The cysteine-rich VARP sequences adopt tightly folded structures that are stabilized by a single Zn^++^ ion. They bind to a hydrophobic pocket on the surface on VPS29, which is freely available in the structure of the assembled retromer coat. We call this structure a Zn-fingernail and show that they appear only in VARP homologues in the lineage directly giving rise to animals Finally we demonstrate that mutations in the Zn-fingernails that abolish VPS29:VARP binding inhibit trafficking from endosomes to the cell surface.

## Results and discussion

### Characterisation of VPS29: VARP CHPLCxCxxC interaction

The two mammalian VARP CHPLCxCxxC sequences differ only slightly in their core sequences but their 20–30 residue flanking sequences differ considerably (Fig. 1b) and are all predicted to be unstructured^26^. Constructs containing the CHPLCxCxxC sequences and their linkers (residues 396–460 and 692–746) were created and a Surface Plasmon Resonance (SPR) assay was developed to study their binding to VPS29 under different conditions (Isothermal Titration Calorimetry (ITC) proved unsuitable primarily due to the constructs’ rapid precipitation on stirring).

Recombinant GST-692-746 and a similarly sized GST control fusion protein were bound to an anti-GST antibody, which had previously been covalently coupled to a sensor chip and untagged VPS29 passed over the resulting surface. The *K*_D_ was measured using both equilibrium and kinetic analysis, both of which yielded a value of 2–3 µM at 12 °C. (Fig. 1c–f). Thermodynamic analysis over a range of temperatures between 8 °C and 25 °C shows that binding has favorable enthalpy and entropy changes (Δ*H*° ~ −20 kJ mol^−^1 and Δ*S*° ~ +36 J K^−1^ mol^−1^) and the reaction is exothermic. When the experiment was repeated using protein that had been dialyzed overnight against buffer lacking Zn^++^ but containing 10 mM EDTA in order to remove bound Zn^++^, and the assay carried out in buffer lacking Zn^++^ and containing 10 mM EDTA, the measured binding dropped ~20 fold to 45 µM (additional unsuitability of ITC resulted from EDTA stripping divalent metal ions from VPS29^27^). A similar weaker *K*_D_ (55 µM) was also obtained in the presence of Zn^++^ ions when all four Cys residues, which we believe coordinate a single Zn^++^, were mutated to serines (4C mutant, Fig. 1e). These data demonstrate a key role for the 4xCys/Zn^++^ cluster in mediating the interaction between VARP and VPS29. Although being more physically unstable and the data consequently of poorer quality, residues 369–460 displayed a similar *K*_D_ to residues 692–746 of ~5μM (Fig. 1e): further study of this sequence was not pursued.

### VPS29: VARP CHPLCxCxxC complex structure

**Fig. 2:**
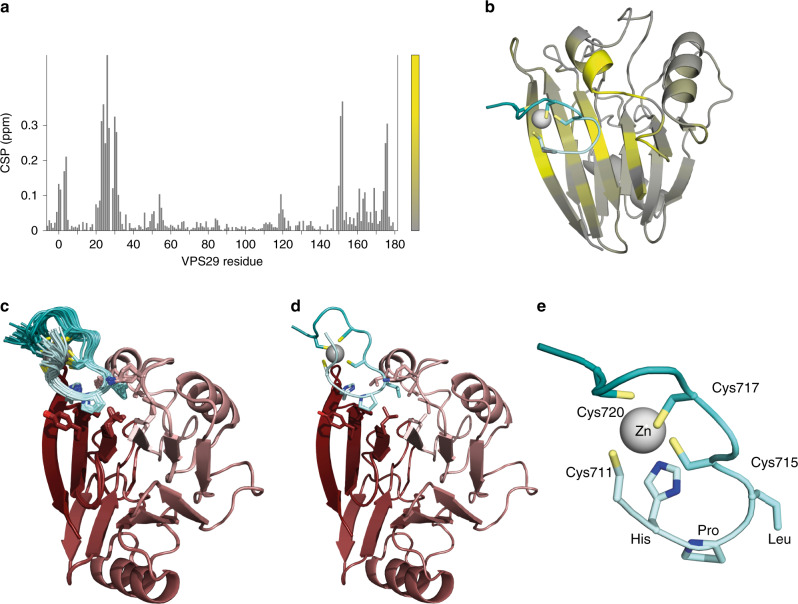
Structure of the VARP 692–746:VPS29 complex. **a** Histogram showing chemical shift perturbation (CSP) values for the amide group signals of ^15^N labelled VPS29 on binding of VARP residues 692–746 calculated according to the equation CSP = (Δδ(^1^H)^2^ + 0.2(Δδ(^15^N)^2^))^0.5^; the largest CSP value is 0.662 (for Leu 26) and is truncated in this plot. The bar to the right of the plot shows the colour code used to map these CSP values to the structure: values between 0 and 0.331 (half the largest CSP value) are shown using a colour ramp running from grey to yellow, while values >0.331 are uniformly shown as yellow. **b** Mapping of the CSP data onto the structural model of the complex of VPS29 with VARP 692–746, using the colour ramp defined in panel a). Only the ordered residues (709–721) of the VARP fragment are shown. **c**, **d** VPS29 coloured from N (pink) to C (dark red), together with the ordered residues (709–721) of the VARP fragment coloured from N (pale cyan) to C (deep cyan) with cysteine side chains shown and the Zn^++^ atom indicated by a grey sphere. **c** shows the ‘family’ of 25 best fit structures of the complex and **d** shows the lowest energy structure. **e** Enlargement of the ordered residues (709–721) of the VARP fragment taken from the lowest energy structure of the complex; key residues are indicated.

The structure of the complex of VARP 692–746 with VPS29 was determined using a hybrid NMR/X-ray crystallographic approach. VPS29 and VARP residues 692–746 were produced in untagged forms as unlabelled, ^15^N-labelled or ^15^N, ^13^C labelled versions. NMR signal assignments for the two free components (Supplementary Fig. 1a, b) and for the 1:1 complex were made using a suite of multinuclear 2D and 3D NMR experiments, in conjunction with samples having different isotopic labelling schemes. Structures were calculated using a combination of inter- and intra-molecular NOE-derived distance constraints for both molecules, J-coupling-derived χ^1^ angle constraints for VARP, and locally adjustable non-crystallographic symmetry (NCS) terms that maintain similarity to a fixed template structure of VPS29 derived from PDB 2R17 (Table 1). This approach is similar to that employed in a recent study of a protein-DNA complex^28^, and builds on previous related approaches for characterizing flexible multi-domain proteins and their complexes^29,30^; in this way we were able to incorporate direct knowledge of the previously known VPS29 domain crystal structure, while our NMR data provided the key information to determine the nature of the interaction with VARP, where flexibility and weak binding pose problems for crystallography. Although an independent structure for VPS29 was not calculated during this study, analysis of secondary chemical shift and CSI (chemical shift index^31^) data showed that the crystal structure of VPS29 is maintained in solution and does not change appreciably upon the formation of the complex with VARP 692–746 (Supplementary Fig. 1c, d), which chemical shift perturbation analysis indicates binds to a patch on the central β-sheet of VPS29 (Fig. 2a, b).

**Table 1:** NMR data collection and refinement statistics for the final set of 25 accepted structures.

	Total	VPS29	VARP
Structural restraints			
NOE-derived distance restraints			
Intraresidue		33	39
Sequential		22	40
Medium (2 ≤ |i-j|≤4)		24	17
Long (|i-j| > 4)		40	2
Total		119	98
Intermolecular	49		
Dihedral angle restraints			
*χ*^1^		None	11
Template non-crystallographic symmetry restraints for VPS29 (to pdb 2R17)			
Backbone			
Strong^a^	Residues 7–19, 60–148		
Medium^b^	Residues 1–6, 20–59, 149–181		
Sidechain			
Medium^b^	Residues 1, 3–6, 20–24, 26, 28–29, 31–59, 149–151, 153, 155–160, 162, 164, 166–171, 173, 175–181		
Weak^c^	Residues 2, 25, 27, 30, 152, 154, 161, 163, 165, 172, 174		
Structural statistics for accepted structures			
Number of accepted structures	25		
Mean XPLOR-NIH energy terms (mean ± s.d., kcal.mol^−1^)			
* E*(total)	−1694.0 ± 23.1		
* E*(van der Waals)	175.8 ± 4.3		
* E*(NCS)^d^	153.8 ± 14.2		
* E*(distance restraints)	39.8 ± 8.3		
Restraint violations (mean ± s.d.)			
Distance (Å)	0.081 ± 0.065		
Dihedral angles (°)	0.99 ± 0.50		
Max. distance restraint violation (Å)	0.646		
Max. dihedral angle violation (°)	2.00		
RMS deviations from the ideal geometry			
Bond lengths (Å)	0.0024		
Bond angles (°)	0.68		
Improper angles (°)	0.51		
Average pairwise atomic rmsd (±s.d.)			
(N, C^α^, C’ atoms)		0.10 ± 0.03 Å^e^	0.33 ± 0.12 Å^f^
(All heavy atoms)		0.21 ± 0.04 Å^e^	0.69 ± 0.15 Å^f^

^a^Force constant 100.0 kcal mol^−1^.

^b^Force constant 2.0 kcal mol^−1^.

^c^Force constant 0.1 kcal mol^−1^.

^d^NCS energy terms are reported for the structures prior to addition of the unstructured tails of VPS29; no NCS terms are active during the final stage of the calculation when these tails are added.

^e^Residues 1–181.

^f^Residues 710–721.

**Fig. 3:**
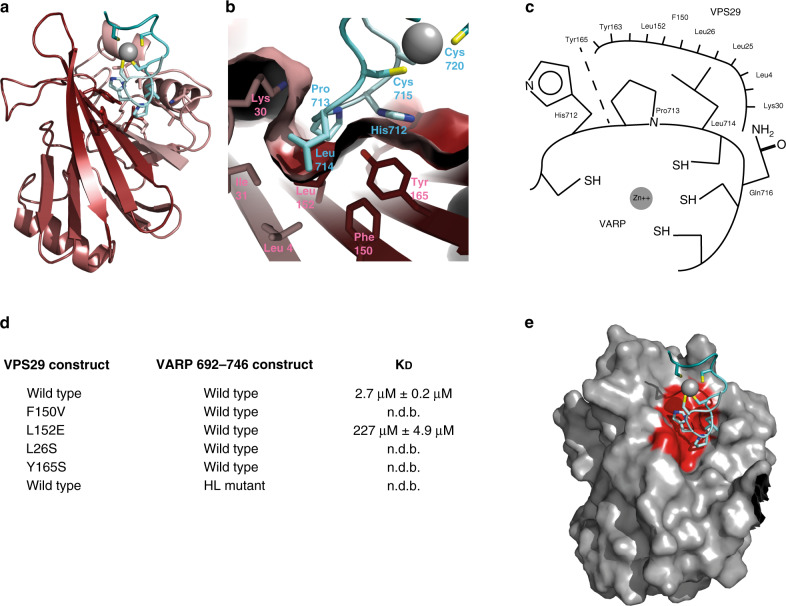
Analysis of VARP residues 692-746:VPS29 interface. **a** View of VARP:VPS29 complex rotated from view in Fig. 2 to better show binding surface. **b** Cut away surface rendering of VPS29 to highlight the hydrophobic pocket in which HisProLeu residues of VARP bind. Key side chains in the interaction are shown and labelled pink (VPS29) or cyan (VARP). **c** Schematic representation of His, Pro, Leu triplet binding to VPS29. **d** K_D_s determined by SPR for mutants in key residues of the VPS29:VARP interface. **e** Same view as (**a**) but shown as surface representation with residues whose mutation abolish binding to residues 692-746 of VARP highlighted in red forming a single shallow cavity on the surface of VPS29.

**Fig. 4:**
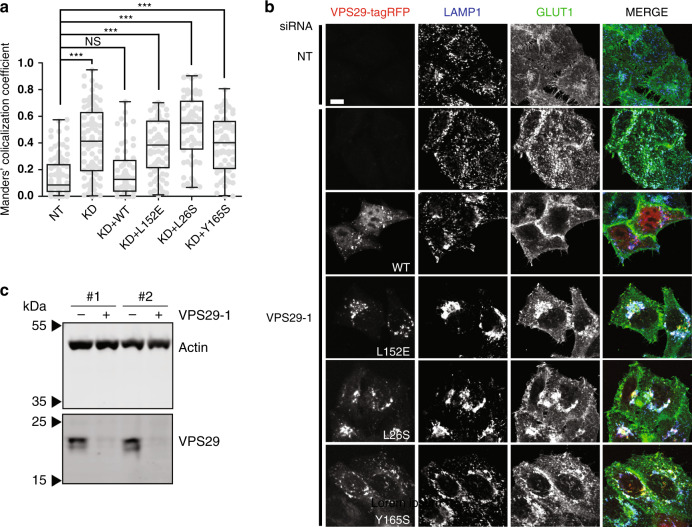
The VPS29-VARP interaction in HeLa cells. **a**–**c** The VPS29-VARP interaction is required for GLUT1 trafficking between endosomes and the cell surface. HeLaM cells knocked down (KD) with VPS29-1 siRNA oligonucleotide or treated with a non-targeting, control oligonucleotide (NT) and, in some cases, transiently expressing VPS29-tagRFP (wt and mutants L152E, L26S and Y165S—all red) were stained with antibodies to LAMP1 and GLUT1 and then imaged by immunofluorescence confocal microscopy. **a** Box and whisker plots showing Manders’ colocalization coefficients for the overlap of GLUT1 with LAMP1. Grey spots show individual cells analyzed from independent experiments. ***p < 0.001; NS, not significant. **b** Representative cell images showing VPS29-tagRFP (red) LAMP1 (blue) and GLUT1 (green). Scale bar 10 μm. **c** Immunoblots showing depletions of endogenous VPS29.

The structure of the complex is shown in Fig. 2c, d. The only residues of VARP for which medium- or long-range restraints could be determined were 711 to 720 (CHPLCQCPKC) (Supplementary Fig. 1e, f). This region forms a tightly folded series of turns about a single Zn^++^ atom (Fig. 2c–e) in agreement with micro PIXE data^19^. The three intervening loops between the cysteines are highly conformationally restricted (Fig. 2e). The loop linking the first two cysteines adopts a specific conformation that presents its His Pro Leu residues (712–714) along with Gln716 to a largely hydrophobic patch formed by side chains from VPS29 Leu4, Leu25, Leu26, Lys30, Leu152, Tyr163 and Tyr165 on VPS29 (Fig. 3). There are also probably contributions to the interface from two well-conserved tyrosine residues on VPS29^32^, which hydrogen-bond to two backbone carbonyl oxygens on VARP: Tyr 165 OH (VPS29) – Pro 713O (VARP) and Tyr 163 OH (VPS29) – Cys 711O (VARP), the latter probably being water-mediated. Simultaneous mutation of the His and Leu residues from the loop in VARP to alanines abolishes binding to wt VPS29 in our SPR assay (Fig. 2e). We had previously postulated a role for VPS29 Leu152 in retromer function^19,27^ when it was fortuitously identified as forming a central part of a highly conserved surface-exposed hydrophobic patch of unknown function in mammalian VPS29, although in yeast Vps29p it appeared to be involved in interacting with a Vps5p/Vps17p dimer^27^. The complex structure presented here provides a mechanistic basis for Leu152’s critical role in binding VARP and its mutation does indeed abolish binding (Fig. 3c, d). Mutations, L26S and Y165S, designed on the basis of the structure likewise abolish binding of VPS29 to VARP residues 692–746 without significantly affecting the folding of VPS29 (on the basis of circular dichroism and incorporation into retromer complexes in vivo—see later and Fig. 4).

The presence of the Zn^++^/4Cys constellation largely fixes the structure of the Zn-fingernail in solution, i.e., it is a conformationally restrained scaffold for displaying the His Pro Leu motif (Figs. 2e and 3c). This reduces the entropic penalty on binding to VPS29 that would occur if the interacting side chains were part of a mobile segment of polypeptide, explaining how the comparatively tight binding seen can be produced from a relatively small buried interaction surface of only ~600 Å^2^ ^33^ (Fig. 3b). In agreement with this, removal of the Zn^++^ or mutating all four cysteines, either of which actions would generate a structurally unconstrained peptide, causes a ~20-fold decrease in binding affinity (Fig. 1e).

### The Zn-fingernail

Zinc fingers are autonomously folding, molecular scaffold domains, in which zinc plays a structural role. They were first identified as DNA binding domains but are now known also to mediate interactions with DNA, RNA or protein. Zn-fingers can be subdivided into eight groups^34,35^. The CHPLCxCxxC motifs resemble none of these to any significant degree: they are considerably smaller than any other reported zinc finger/zinc knuckle domain, being only ten residues in length; have no secondary structural elements; contain no hydrophobic core formed by residues between the cysteines; and are not buried or tightly associated with a protein surface but will instead protrude ~60 Å on ~20 residue unstructured linkers from the main body of VARP. We have therefore named this structure a Zn-fingernail.

Interrogation of the PDB found only a few autonomously folding microdomains of a similar size, none of which were metal-ion binding and none were of a similar structure. ALEPH software^36^ indicates that there are no regions of any known structure’s peptide backbone that have an r.m.s.d. less that 1.6 Å with the backbone of the Zn-fingernail and in those closest in structure to the Zn-fingernail, several of the side chains always point in very different directions and none bind metal ions. ALEPH also indicates that there are no structures in the PDB containing the pattern of four cysteines in CHPLCxCxxC that bear any structural resemblance to the Zn-fingernail. In bioinformatic searches of the existing non-redundant protein sequence database using CHPLCxCxxC, we did not detect any other meaningful sequence matches other than in VARP orthologues, although using the conserved pattern of cysteines alone revealed several hits corresponding to parts of larger multiple metal ion binding proteins including FeS centres and Metallothioneins. Hence, to the extent that primary sequence reflects secondary structure, it appears that the Zn-fingernail is itself unique and is unique to VARP.

### The VPS29:VARP interaction in vivo

When transiently expressed in HeLa cells, VPS29-TagRFP harbouring mutations in the Zn-fingernail:VPS29 interface L26S and Y165S as well as L152E all colocalized with retromer (VPS35) on endosomes similarly to wt VPS29-TagRFP, indicating that they were correctly folded (Supplementary Fig. 2a). However, all three of the mutations that abolished the interaction of VPS29 and VARP in vitro resulted in only cytosolic localization of VARP-GFP, which was lost upon cytosol extraction (by saponin treatment prior to fixation of the cells; Supplementary Fig. 2b). Trafficking of the transmembrane protein GLUT1 between endosomes and the cell surface can be used as a measure of retromer function^19,20,37^. Depleting VPS29 in HeLa cells resulted in increased colocalization of GLUT1 and the late endosomal/lysosomal membrane protein LAMP1 ((Fig. 4) and ref. ^19^), consistent with a block in recycling of GLUT1 from endosomal compartments to the plasma membrane. This could be rescued in transiently transfected HeLa cells expressing wtVPS29-tagRFP but not by the L26S, Y165S and L152E mutant versions of VPS29-tagRFP (Fig. 4b).

The lack of rescue by the L152E mutant was consistent with our previous data^18^, but not with the subsequent observation by Jimenez-Orgaz et al.^15^, that expression of the L152E mutant was as effective in rescuing GLUT1 recycling as wild type protein in a VPS29 knockout cell line. In the course of these experiments, we noticed a subtle change in the pattern of some endosomal/lysosomal markers when expressing the L26S, Y165S and L152E mutant versions of VPS29-tagRFP but not wtVPS29-tagRFP (see LAMP1 in Fig. 4b, VPS35 and VARP-GFP in Supplementary Fig. 2b). A likely explanation is the inability of the VPS29 mutants to bind TBC1D5, since it has previously been observed that changes to endosome clustering and morphology occur when expressing the catalytically inactive TBC1D5 R169A/Q204A mutant (see Fig. 1 in ref. ^38^). We did not investigate further as the main focus of this work is not on TBC1D5.

The interaction of VARP with VPS29 is highly unusual, possibly unique, amongst transport coat protein networks, in that although mediated by a short linear motif with a folded domain as is normal (see ELM database elm.eu.org), the motif is actually part of a structurally defined fold (the Zn-fingernail) resulting in a decrease in entropy loss and hence relatively tight binding of 2–3 μM for the motif when compared with similar motifs such as Asp Pro Phe, Asp Pro Trp (binding AP2 α-adaptin appendage) and Asn Pro Phe (binding EH domains) all of which consist of a proline flanked by a hydrophilic and a hydrophobic residues and have K_D_s in the 100 μM range. Notably, conformational restraining of an Asn Pro Phe motif with a non-physiological disulphide link causes a ~10 fold reduction for its cognate EH domain in K_D_ to ~10 μM^39,40^. Similarly, removal of the Zn^++^ ion destabilizes the VARP Zn-finger domain, thereby reducing its affinity to VPS29. However, it is possible to regain this affinity by adding back Zn^++^ ions since in bio-layer interferometry it was shown that GST VARP 692–746 (fingernail 2) bound VPS29 prior to the EDTA treatment and following EDTA treatment and subsequently refolding into buffer containing Zn^++^ with similar affinities (Supplementary Fig. 3a).

### Comparison with other VPS29 binding partners

Whilst this study was underway, X-ray crystallography structures of VPS29 and VPS29/VPS35 C-terminal domain complexed with different ligands (legionella infectivity factor RidL (PDB IDs 5WYH and 5OSI) and a small peptide fragment of the endosomal Rab7 GAP TBC1D5^16,41^ (PDB ID 5GTU)) were reported^42–45^. Notably, these studies used a slightly longer isoform of VPS29 that has the N-terminal sequence MAGHRLVLVL (referred to here as long VPS29) instead of MLVL; the latter was the originally reported isoform^46^ and is the one used in this work. Importantly, the affinities of either VPS29 isoforms for VARP were similar: *K*_D_s were 1.3 and 2.7 µM respectively, with equivalently fast on-rates (Supplementary Fig. 3b).

**Fig. 5:**
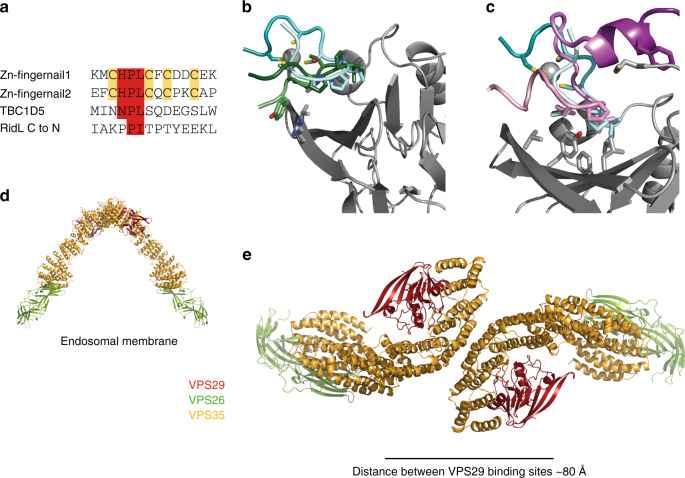
Ligand binding to VPS29 in context of whole retromer complex. **a** Structure-based alignment of VPS29 binding sequences from vertebrate VARP fingernails, TBC1D5 and Legionella RidL (C to N direction as in peptide complex structures). The key binding residues are boxed in red and the cysteines in VARP boxed in yellow. **b** Superposition of RidL peptide ligand (green) with VARP Zn-fingernail (N pale cyan) to C (dark cyan) binding at the same hydrophobic site (PDB ID 5WYH). **c** Superposition of TBC1D5 peptide (PDB ID 5GTU) N (pale purple) to C (dark purple) bound to longer N-terminal isoform of VPS29 and VARP Zn-fingernail (N pale cyan to C dark cyan). The ligands bind to the same site on VPS29 with the Pro and Leu residues from both superimposing well. The extended VPS29 N-terminus including the important binding residues -3 to 0 pack against an α-helix from TBC1D5. **d**, **e** views of a retromer coat arch formed by two VPS26(green)/Vps29(red)/VPS35(gold) heterotrimers shown parallel to (**d**) and looking down towards (**e**) the endosomal membrane. The distance between VPS29 binding sites is ~80 Å allowing the Zn-fingernails from one VARP to easily bind to a single arch.

Both RidL and TBC1D5 bind to the same site on VPS29 as do the VARP Zn-fingernails using proline and leucine/isoleucine residues, but neither possesses a Cys/Zn^++^ cluster (Fig. 5a–c). Binding of the legionella infectivity factor RidL does not involve the additional MAGHRL residues and has a comparatively high affinity (K_D_ 200–400 nM) due to a large total interaction interface of 850–920 Å^2^, consistent with its ability to outcompete VARP and TBC1D5 binding^42–45^ (Fig. 5b). The MAGHR residues however, are involved in binding of TBC1D5 to VPS29 (Fig. 5c). A short α-helix formed by residues upstream of the critical Asn Pro Leu motif in TBC1D5 provides a major part of the binding interface, packing against MAGHR^43^. Using our SPR assay (Supplementary Fig. 3b), we measured a *K*_D_ ~67 µM for the affinity of TBC1D5 (132–156) to the long VPS29, which is weaker than has been reported by ITC (residues 132–158: ~20 µM^43^). The interaction of short VPS29 lacking MAGHR was too weak to assess reliably (*K*_D_ > 300 μM) likely due to factors including the interaction being mediated only by the Asn Pro Leu of TBC1D5 (analogous to His Pro Leu in VARP (Fig. 5a)) contacting the Leu152-centred hydrophobic patch and the entropic penalty resulting from the absence of the conformationally restrictive 4Cys:Zn^++^ cluster present in VARP. However, the overall affinities of TBC1D5 for the retromer core complex containing either form of VPS29 will be enhanced by simultaneous binding to VPS35^42–45^ due to avidity effects. Tighter binding to VPS29 would likely render TBC1D5 constitutively attached to retromer, and this does not appear to be the case as some retromer positive tubes are TBC1D5 negative; VARP can readily compete with TBC1D5 for retromer binding (see next section); and Rab7:GTP-dependent retromer recruitment^16^ would be difficult as the GTP on the Rab7 would be immediately hydrolysed. Use of different relative amounts of the two versions of VPS29, for instance in different cell types, could bias the ratio of VARP to TBC1D5 in a given retromer coat, with various physiological outcomes, e.g., amount of Rab7 or VAMP7, resulting (see later).

### VARP and TBC1D5 binding in the context of retromer assemblies

One Zn-fingernail can be accommodated on the surface of one Leu152-centred binding site on VPS29 (Fig. 3a, b). The recent cryo-electron tomography structures of *Chaetomium thermophilum* and *Chlamydomonas reinhardtii* retromer coats reveals the presence of VPS35 dimers forming ~100 Å high arches extending away from the membrane with two VPS29 molecules placed at each apex^47^ (Fig. 5d). The existence of arches has also been confirmed by SAXS and electron microscopy^48–50^. On a membrane, the ~80 Å separating the L152-centred binding sites on the VPS29s in a single arch (Fig. 5d) would easily allow simultaneous binding of the two Zn^++^-fingernails protruding on their unstructured linkers from the starts of the two Ankyrin repeat domains of a single VARP molecule. Thus, by avidity effects, a VARP molecule will prefer to bind simultaneously to adjacent VPS29’s in a coat-assembled arch over isolated retromer VPS26/VPS29/VPS35 heterotrimers. As well as binding to VPS29, VARP may also bind weakly to retromer via VPS35^20^ (the two possibilities are not mutually exclusive).

**Fig. 6:**
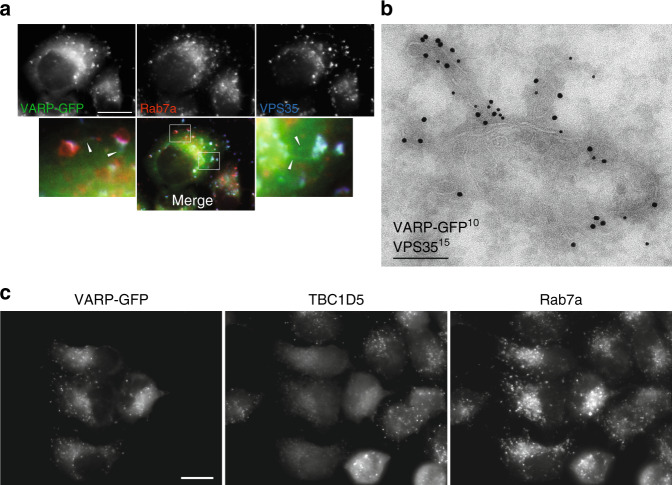
VARP is enriched on endosomal tubular/vesicular carriers. **a** Immunofluorescence wide field microscopy images of HeLaM cells stably expressing VARP-GFP (green) showing colocalization with Rab7a (red) and VPS35 (blue) on puncta and tubules (arrow heads). Scale bar 20 μm. **b** Immunoelectron micrograph showing colocalization of VPS35 and VARP-GFP on tubules emanating from a multivesicular body in a HeLaM cell stably expressing VARP-GFP. Scale bar, 200 nm. **c** Immunofluorescence wide field microscopy images of a mixed population of HeLaM cells, some stably expressing VARP-GFP. Cells expressing VARP-GFP show a reduction in punctate labelling of TBC1D5 and increase in labelling of Rab7a. Scale bar 20 μm.

In line with this, using immunofluorescence microscopy VARP is seen on retromer and Rab7 positive endosomes and tubular structures protruding from them (Fig. 6a) and refs. ^16,19^). Immunoelectron microscopy, which allows short, retromer- and VARP-positive tubules to be resolved from the endosomal body from which they emanate, suggests that the retromer and VARP are enriched on the tubular processes (Fig. 6b), where the retromer will be anchored through coincidence detection with cargo and SNX proteins^51^ and the VARP through binding to retromer arches. The presence of VARP in these retromer carriers will facilitate both the incorporation of VAMP7 into the carriers as well as the generation of Rab21:GTP on them for their subsequent docking and fusion to their target membranes^21,22,24^. The presence of VARP on the main bodies of endosomes (Fig. 6) can be accounted for by its ability to bind the late endosomal Rab family member, Rab32:GTP^19,23^, to free endosomal VAMP7 and likely also to ‘unpolymerised’ membrane-attached, VPS26/VPS29/VPS35 heterotrimers.

The Rab7 GAP TBC1D5 is also a ligand of retromer, but will have little preference for arch-assembled retromer over unpolymerised cytosolic or membrane-associated retromer as it binds to both VPS29 and VPS35 simultaneously in the same retromer heterotrimer^43^. By immunofluorescence microscopy TBC1D5 is found on retromer-positive endosomes and some of their tubular projections (Supplementary Fig. 3c)^16,19^. Mild ectopic over-expression of VARP-GFP such that the amount of VARP-GFP + VARP in the mixed stable cell population is <2 fold that of VARP alone in untransfected cells, causes TBC1D5 to be largely displaced from endosomal membranes with a concomitant increase in levels of membrane-attached Rab7 (Fig. 6c) due to removal of the TBC1D5’s Rab7 GAP activity, consistent with a competition between the two proteins for the VPS29-binding pocket (Fig. 5c). This competition occurs despite there being <50-fold excess of retromer core subunits over both VARP and TBC1D5^52^, which implies that there should be many VPS29 proteins that are ligand-free. One possible explanation is that the in vivo situation may thus be more complex than current models suggest: nevertheless, the data presented here show that VARP can compete with TBC1D5 in vivo for the pool of retromer that is assembled into a coat on endosomes. Regardless of precise mechanism, these data indicate that the ratio of bound VARP vs. TBC1D5 is finely balanced such that even a slight perturbation can cause an obvious in vivo effect. In this context, it is worth noting the differences amongst previous reports with regard to whether modulation of TBC1D5 has an effecting on retromer-dependent recycling of plasma membrane proteins. Whereas both we^18^ and Jimenez-Orgaz et al.^15^ found no effect of depleting TBC1D5 in mammalian cells on GLUT1 recycling, an effect on integrin recycling has been reported^40^ and the effect of modulating autophagy on GLUT1 recycling has been proposed to be mediated via redistribution/shuttling of TBC1D5^53^ In addition, both VPS29 and TBC1D5 have been implicated in synaptic vesicle recycling and synaptic transmission in *Drosophila*, an experimental model in which, as in our experiments, only wild type and not the L152E mutant of VPS29 was able to rescue the effect of VPS29 knockout^54^.

**Fig. 7:**
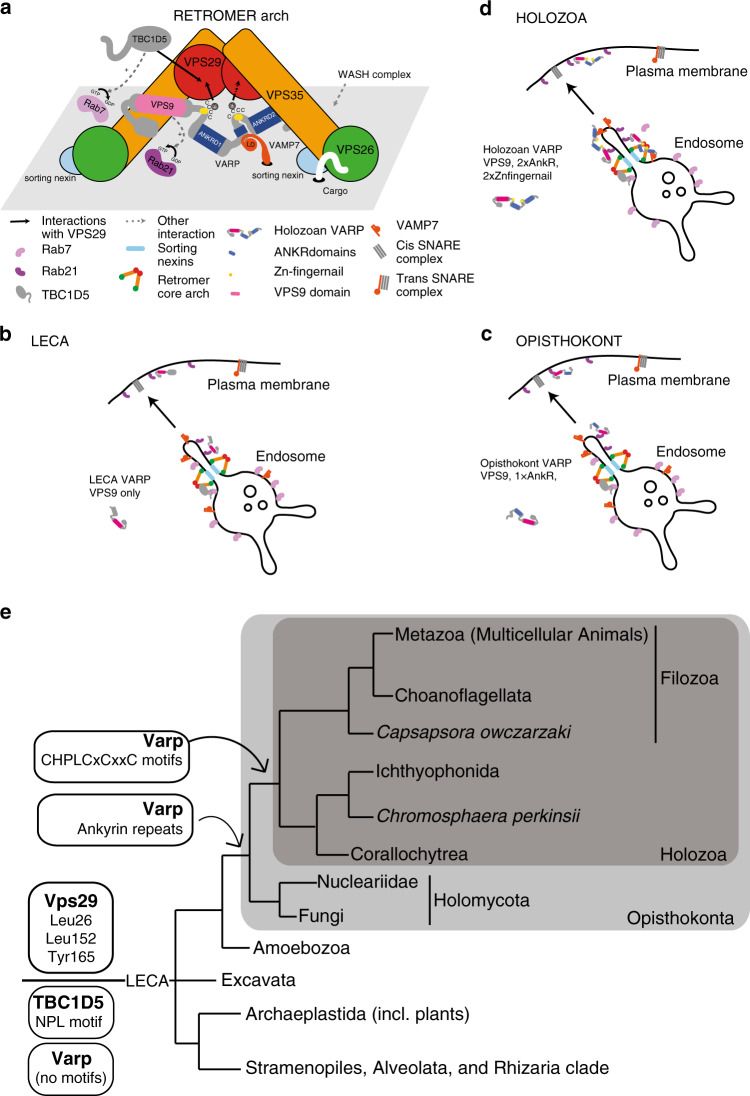
Model and evolution of the VPS29/VARP/TBC1D5 interactions. **a** Model depicting possible assemblage of VARP, TBC1D5 and their functional partners onto endosomal membrane-attached retromer arches. **b**–**d** Hypothesized cellular configuration of VARP, TBC1D5, and VPS29 and associated endosomal partners (coloured as in key) in three reconstructed ancestral nodes. Reading anticlockwise, in the LECA (**b**), Retromer interacts with Rab7 mediated by TBC1D5, while Varp interacts with Rab21 at the endosome and Plasma membrane. In the opisthokont ancestor (**c**), the addition of the ankyrin repeats allows for possible interaction of Varp with Vamp7. By the time of the holozoan ancestor (**d**), all of the relevant domains (including the two Zn-fingernails) had been added to VARP to compete for Vps29 with TBC1D5 using the same conserved motif providing for direct interaction with retromer. **e** Proposed origin timing of the VPS29 Leu26, Leu152, and Tyr165 residues, the VARP Zn-fingernail, and the TBC1D5 AsnProLeu motif in eukaryotes. This schematic of eukaryotic relationships, with emphasis on the lineages leading to metazoan shows the acquisition points of proteins (bold) and motifs/residues at relevant nodes. See Supplementary Fig. 4 for supporting alignments.

Access of the VPS9 ANKRD1 (Rab21GEF) and the ANKRD2 domains of VARP to membrane-associated VAMP7 and Rab21 will be facilitated by the unstructured (lack of medium or long range restraints (Supplementary Fig. 1c, d) and a lack of predicted secondary structure^26^) linkers on the Zn-fingernails. Efficient VARP recruitment into retromer-based coats is easiest to envisage occurring as the retromer coat assembles: either if the VARP is already dynamically localized to the endosome surface through transient interactions with its membrane-associated binding partners and it ‘reaches up’ to the arches or if it docks from the cytosol and ‘reaches down’ to its partners (Fig. 7a).

### Molecular evolution of the VPS29 interaction platform

VARP, VPS29 and TBC1D5 are all ancient proteins, present in the Last Eukaryotic Common Ancestor^5,55,56^. However, co-occurrence need not necessarily indicate interaction. Orthologues of each protein were sampled across the relevant taxonomic range (Fig. 7, Supplementary Fig. 4, Supplementary Table 1) and examined for the presence of the key biochemical determinants identified above, enabling an evolutionary reconstruction of the VPS29 interaction platform.

While VARP orthologues are present across eukaryotes, the Zn-fingernails and the stacked Ankyrin repeats, the latter binding VAMP7 and Rab32 are not^56^. Some fungal ‘VARP proteins’ possess a single set of ankyrin repeats but without the Zn-fingernail C-terminal to the VPS9 domain, suggesting that this domain organization existed in the VARP orthologue present in the ancestor of animals and fungi (i.e. the Opisthokonta) (Fig. 7, Supplementary Fig. 4). Nonetheless, VARP sequences from *Salpingoeca rosetta* and *Capsaspora owzarzaki* possess the double Zn-fingernail-Ankyrin Domain architecture, suggesting that this was already present in the ancestor of the Holozoa (Fig. 7, Supplementary Fig. 4). In VPS29 proteins, the critical VARP and TBC1D5 binding residues Leu26 and Leu152, are highly conserved in diverse eukaryotes (Supplementary Fig. 4). Finally, in TBC1D5, the VPS29 binding Asn Pro Leu motif is also relatively well conserved (occasionally His Pro Leu or Asp Pro Leu (Supplementary Fig. 4) across eukaryotes, and so these three last biochemical determinants can be reconstructed as present in LECA.

We therefore propose an evolutionary model for the VARP-TBC1D5-Retromer regulatory network (Fig. 7b–e). In LECA (Fig. 7b), VPS29 likely interacted exclusively, albeit weakly, with TBC1D5 that acted as the Rab7GAP. Rab7 and VAMP7 were present in LECA^57–59^, the former acting in late endosomal function and thus being inactivated by TBC1D5, and the latter likely acting to promote fusion of the tubule with the plasma membrane. A VARP-like protein would have acted as a GEF on Rab21, on internal membranes and the plasma membrane but likely independently from retromer^60^. In the opisthokont ancestor (Fig. 7c), however, a single set of Ankyrin repeats had been acquired in VARP, which allows for direct interaction with VAMP7, and potentially indirectly with retromer. Between the opisthokont and holozoan ancestor, the Zn-fingernail was acquired and the Zn-fingernail-Ankyrin domain unit most likely duplicates, establishing the modern holozoan and animal configuration of VARP (Fig. 7d).

By providing selective pressure to fix the relevant residues in VPS29, the VPS29:TBC1D5 interaction from the LECA onward essentially facilitated the much later emerging VPS29:VARP interaction at the Zn-fingernails, since only the relevant residues in VARP would need to evolve for the interaction to evolve. This sets up potential competitive binding between VARP and TBC1D5 demonstrated in vivo in the previous section, which in turn could influence retromer-based carrier generation dynamics and thus be a pre-adaptation for the development of complex endosomal recycling pathways in multicellular animals. In vertebrates, the binding of TBC1D5 is enhanced by the evolution of the long splice form of VPS29, potentially shifting the balance of binding to TBC1D5 again.

In summary, in mammalian cells, a VARP molecule can interact with two VPS29’s, likely on a single retromer arch through insertion of an [His Asn Asp]Pro Leu motif, whose presentation is defined by a unique 10 residue Zn^++^-stabilised structure, into a hydrophobic pocket on VPS29. VARP and TBC1D5, which can compete for binding to the same site on VPS29, play key roles in coordinating endosomal recycling dynamics in animal cells by integrating Rab21 and Rab7 function to generate an appropriate Rab complement on retromer-coated tubes so as to prepare them for fusion with/integration into the plasma membrane or earlier endosomes^61–63^. VARP and TBC1D5 do this by binding to a common site on VPS29 that likely arose initially for TBC1D5 binding. Over the course of the lineage that gives rise to animals and fungi, and then animals themselves, VARP began interacting increasingly with members of the retromer regulatory network, reaching the highly integrated state observed today. VARP additionally affects trafficking within the endocytic system through being an effector for Rab32^19,23,51^ and ensures efficient recruitment of a SNARE, VAMP7, into retromer-based carriers^21,22,25^ that can drive their ultimate fusion with their target membranes. Understanding the molecular basis of VARP’s binding to retromer is key to understanding this protein’s role in membrane trafficking.

## Methods

### Protein expression and purification

All constructs used are summarised in Supplementary Table 1. Zn fingernails were expressed as N-terminal, prescission-cleavable GST fusion protein from pGEX6P1 in BL21 (DE3) pLysS E. coli Expression was carried out in media supplemented with 0.5 μM ZnCl_2_ at 22 °C for 16 h following induction with 0.2 mM IPTG at OD_600nm_ of 0.8. For expression of isotopically labelled proteins 2TY 10 mL starter cultures at OD_600nm_ of 0.8 were pelleted at 4000 rpm for 20 min and resuspended in 1 L M9 minimal media (enriched only with ^15^NH_4_Cl (0.5 g/L) or with both ^15^NH_4_Cl (0.5 g/L) and ^13^C_6_-glucose (2 g/L)) (and, 0.5 μM ZnCl_2_ for Zn-fingernails). Expression was carried out for 18–20 h at 28 °C following induction of expression by addition of 0.2 mM IPTG at OD_600nm_ of 0.8.

Proteins were purified in 20 mM Tris, 100 mM NaCl, pH7.4, 0.2 mM βME, 0.2 mM AEBSF on GST-Sepharose. Prescission cleavage was carried out overnight at 4 °C if needed with 0.5 mg Prescission Protease. Eluted proteins were further purified on S75 Superdex gel filtration in the same buffer. Samples for NMR were subsequently buffer exchanged into 20 mM ^2^H_11_ Tris buffer pH 7.0, 200 mM NaCl, 1 mM ^2^H_6_ DTT either in a 95:5 H_2_O:^2^H_2_O mixture or in ^2^H_2_O, using a VivaSpin 3000 mwco spin filter. In the latter case, buffer exchange was repeated five times with a 3–5 fold dilution so as to reduce the level of H_2_O from the original buffer to <0.5%. VPS29 was expressed from pGEX4T2 and purified as described above except that cleavage was carried out with thrombin.

### SPR analysis

Experiments were performed on a Biacore T200 (GE Healthcare Life Sciences). In the first instance, goat anti-GST polyclonal antibody was coupled to Series S CM5 Sensor Chips (GE Healthcare Life Sciences, catalog no. BR-1005-30) by the amine coupling protocol specified in the Biacore T200 Control Software with reagents purchased from GE Healthcare Life Sciences. In brief, after the sensor surfaces were activated by applying a 1:1 mixture of 50 mM N-hydroxysuccinimide (NHS): 200 mM 1-ethyl-3-(3-dimethylaminopropyl) carbodiimide hydrochloride (EDC) provided in the Biacore Amine Coupling Kit (GE Healthcare Life Sciences, catalog no. BR-1000-50), the anti-GST antibody was diluted to 30 μg/ml in 10 mM sodium acetate, pH 5.0 (GE Healthcare Life Sciences, catalog no. BR-1003-49) and injected into all flow cells at 5 μl/min for 7 minutes, (resulting in ~10000RU (resonance units) of protein immobilized). Ethanolamine (1 M, pH 8.5) was then injected to block remaining active sites on all four flow cells. In the next step, in order to block any high affinity sites that are present on the anti-GST pAB, 3 cycles using a 3-min injection of recombinant GST at 5 μg/ml followed by regeneration with 10 mM glycine-HCl pH 2.1 were performed.

The binding analysis was performed between 8 and 25 °C in VPS29 gel filtration buffer (20 mM Tris, 200 mM NaCl_2_, 0.1 mM TCEP, 0.5 μM ZnCl_2_, pH7.4). Two flow cells could be analysed at a time. Initially, GST-tagged proteins are injected separately into each flow cell, at 10 μg/ml at 15 μl/min for 200 s. Therefore, the blank flow cell contained GST-CLA and the second flow cell displayed GST-VARP Zn-fingernail, its mutants or GST-TBC1D5 (residues 132–156). GST-CLA is derived from the β2 adaptin subunit of human AP2 (UniProt P63010), residues 623–632, and therefore would not bind VPS29 specifically. After a stabilization period of 300 s, analytes typically between 40 and 0.3μM concentration were then injected at 10 μl/min for 300 s, followed by 300 s wash (dissociation period). Importantly, fast on and off binding events to the different GST-Zn-fingernails, as well as the GST-TBC1D5 (residues 132–156) were observed. Between each sample injection, the anti-GST antibody on the chip was regenerated by injecting 10 mM glycine-HCL pH2.1 at 30 μl/min for 60 s. At 12 °C, each flow cell could be efficiently regenerated ~50 times.

Sensorgrams were processed with Biacore T200 Evaluation Software (ver. 2.0.1, GE Healthcare Life Sciences). Equilibrium dissociation constants (*K*_D_) for binding were determined from the best fit curves to the equation for a single binding site between the VARP Zn-fingernail and VPS29. On and off-rates, and resultant *K*_D_, were also calculated, using *R*_max_ determined from the equilibrium analysis. By acquiring binding data at five different temperatures (between 4 and 25 °C), the Van ‘t Hoff equation was plotted to estimate changes in binding enthalpy and entropy. The linear form of the Van ‘t Hoff equation:

1 $$\ln {K}_{\mathrm{D}} = \frac{{{\Delta}{H}}}{{{R}.{T}}} - \frac{{{\Delta}{S}}}{{R}}$$

(where *R* is the gas constant, and Δ*H* and Δ*S* are the binding enthalpy and entropy, respectively) was used, due to the clear linearity of ln *K*_D_ plotted against 1/T in that temperature range (*R*^2^ = 0.91). The equation assumes that the enthalpy and entropy are constant with temperature changes. Under standard conditions, at 25 °C, the binding is therefore characterized by favorable enthalpy and entropy changes (Δ*H*° ~ −20 kJ mol^−1^ and Δ*S*° ~ +36 J K^−1^ mol^−1^).

### Bio-layer interferometry experiments

Real-time kinetic measurements of VPS29 binding to immobilized GST-VARP-692-746 at 25 °C were conducted using an Octet RED96 (Pall FortéBio). Samples and buffers were dispensed into 96-well microtiter plates (Greiner) at a volume of 200 µl per well. Anti-GST biosensors (Pall FortéBio) were hydrated in VPS29 gel filtration buffer (200 mM NaCl 20 mM Tris pH 7.4 0.1 mM TCEP 0.5μM ZnCl_2_) for at least 30 minutes prior to loading with GST-VARP-692-746 for 10 minutes. Every binding experiment consisted of three steps: incubation for 10 minutes in the specific assay buffer (200 mM NaCl 20 mM Tris pH 7.4 0.1 mM TCEP with either 0.5μM ZnCl_2_ or 10 mM EDTA) followed by 5 min incubation with VPS29 in the same buffer (8.88–0.14 μM, twofold dilution series) (association phase), and then by 10 min incubation with the same buffer without VPS29 in order to measure VPS29 off-rate (dissociation phase). Three sequential binding experiments were therefore performed on each biosensor tip presenting GST-VARP-692-746: the first in the presence of Z^++^ ions, the second after removal of Zn++ ions (through the use of 10 mM EDTA), and the third in the presence of re-introduced Z++ ions. Importantly, in order to remove Zn^++^ ions, the biosensor-bound GST-VARP was incubated twice for 15 minutes in (200 mM NaCl 20 mM Tris pH 7.4 0.1 mM TCEP 10 mM EDTA). After binding in the presence of EDTA, the biosensor-bound GST-VARP was reloaded with Zn^++^ for 30 min in (200 mM NaCl 20 mM Tris pH 7.4 0.1 mM TCEP 0.5 mM ZnCl_2_). Data were processed with the Octet Data Acquisition 7.0 software, individually fitted using ForteBio Analysis Software 7.0, and subsequently further analysed with the PRISM software (GraphPad) in order to determine equilibrium dissociation constants (*K*_D_). Although VPS29 binding in the presence of 10 mM EDTA was observed to be non-specific and random, both binding experiments in the presence of 0.5 μM Zn^++^ gave similar binding profiles and equilibrium constants.

### NMR spectroscopy

NMR samples of VPS29 comprised 0.3–0.6 mM ^15^N- or ^15^N,^13^C-labelled protein solutions in 20 mM ^2^H_11_ tris buffer pH 7.0, 200 mM NaCl, 1 mM ^2^H_6_ DTT in 95:5 H_2_O:^2^H_2_O. NMR samples of VARP 692–746 comprised 0.3–1 mM ^15^N- or ^15^N,^13^C-labelled protein solutions in 20 mM ^2^H_11_ Tris buffer pH 7.0, 200 mM NaCl, 1 mM ^2^H_6_ DTT in 95:5 H_2_O:^2^H_2_O. In addition to the native VARP residues 692–746, the sequence studied here carried a non-native N-terminal sequence GPLGS (here numbered 687–691) and a non-native Trp residue (here numbered 747). Similarly, the VPS29 samples carried an additional non-native N-terminal sequence GSPEFGTRDR, here numbered -9-0. Samples of complex were prepared by mixing the required amounts of appropriately labelled VPS29 and VARP 692–746. NMR data were acquired using Bruker AV-I 600 and AV-III 800 spectrometers, each equipped with a cryogenically cooled triple resonance (^1^H/^15^N/^13^C) 5 mm probe. Experiments were conducted at 25 °C unless otherwise stated, and ^1^H chemical shifts were calibrated using sodium 3,3,3-trimethylsilylpropionate (TSP) as an external ^1^H reference; 15N and 13C chemical shifts were indirectly referenced to the ^1^H shifts using the ratio of gyromagnetic ratios^31^

For H_2_O samples of ^15^N,^13^C-labelled free VSP29 the following datasets were acquired: 2D datasets: [^15^N-^1^H] HSQC, [^13^C-^1^H] HSQC (aliphatic region in ^13^C), [^13^C-^1^H] HSQC (aromatic region in ^13^C), constant-time [^13^C-^1^H] HSQC (aliphatic region in ^13^C), constant-time [^13^C-^1^H] HSQC (aromatic region in ^13^C); 3D datasets: CBCANH, CBCA(CO)NH, HNCA, HN(CO)CA, HBHANH, HBHA(CO)NH, [^1^H-^13^C-^1^H] HCCH-COSY, [^1^H-^13^C-^1^H] HCCH-TOCSY, [^13^C-^13^C-^1^H] HCCH-TOCSY, ^15^N NOESY-HSQC (τ_m_ = 150 ms), ^13^C NOESY-HSQC (τ_m_ = 150 ms; aliphatic region in ^13^C). For ^2^H_2_O samples of ^15^N,^13^C-labelled free VSP29 the following datasets were acquired: 2D datasets: [^1^H-^1^H] DQ correlation; 3D datasets: ^13^C NOESY-HSQC (τ_m_ = 150 ms; aromatic region in ^13^C).

For H_2_O samples of ^15^N,^13^C-labelled free VARP 692–746 the following datasets were acquired: 2D datasets: [^15^N-^1^H] HSQC, [^13^C-^1^H] HSQC (full spectral width in ^13^C), constant-time [^13^C-^1^H] HSQC (aliphatic region in ^13^C), constant-time [^13^C-^1^H] HSQC (aromatic region in ^13^C), [^1^H-^1^H] NOESY (τ_m_ = 150 ms); 3D data sets: CBCANH, CBCA(CO)NH, HBHANH, HBHA(CO)NH, [^1^H-^13^C-^1^H] HCCH-COSY, [^1^H-^13^C-^1^H] HCCH-TOCSY, [^13^C-^13^C-^1^H] HCCH-TOCSY, HACAHB, HNHB, ^15^N NOESY-HSQC (τ_m_ = 50 ms), ^15^N NOESY-HSQC (τ_m_ = 150 ms), ^13^C NOESY-HSQC (τ_m_ = 150 ms; aliphatic region in ^13^C), ^13^C NOESY-HSQC (τ_m_ = 150 ms; aromatic region in ^13^C).

For H_2_O samples of complex containing 1:1 ^15^N,^13^C-labelled VSP29 and natural abundance VARP 692–746, the following datasets were acquired: 2D datasets: [^15^N-^1^H] HSQC, [^13^C-^1^H] HSQC (aliphatic region in ^13^C), [^13^C-^1^H] HSQC (aromatic region in ^13^C), constant-time [^13^C-^1^H] HSQC (aliphatic region in ^13^C), [^1^H-^1^H] NOESY (τ_m_ = 150 ms, with filter elements set to reject protons coupled to ^13^C or ^15^N in F_1_ and to accept protons coupled to ^13^C or ^15^N in F_2_); 3D datasets: HNCA, HN(CO)CA, CBCANH, CBCA(CO)NH, HBHA(CO)NH, [^1^H-^13^C-^1^H] HCCH-TOCSY, [^13^C-^13^C-^1^H] HCCH-TOCSY, ^15^N NOESY-HSQC (τ_m_ = 150 ms), ^13^C NOESY-HSQC (τ_m_ = 150 ms; aliphatic region in ^13^C), ^13^C NOESY-HSQC (τ_m_ = 150 ms; aromatic region in ^13^C). For ^2^H_2_O samples of complex containing 1:1 ^15^N,^13^C-labelled VSP29 and natural abundance VARP 692–746, the following datasets were acquired: 2D datasets: [^13^C-^1^H] HSQC (aliphatic region in ^13^C), [^13^C-^1^H] HSQC (aromatic region in ^13^C), constant-time [^13^C-^1^H] HSQC (aliphatic region in ^13^C), constant-time [^13^C-^1^H] HSQC (aromatic region in ^13^C), [^1^H-^1^H] NOESY (τ_m_ = 150 ms and 70 ms, with filter elements set to reject protons coupled to ^13^C or ^15^N in F_1_ and to accept protons coupled to ^13^C or ^15^N in F_2_); 3D datasets: [^13^C-^13^C-^1^H] HCCH-TOCSY, ^13^C NOESY-HSQC (τ_m_ = 50 ms and 120 ms; aliphatic region in ^13^C), ^13^C NOESY-HSQC (τ_m_ = 50 ms and 120 ms; aromatic region in ^13^C), ^13^C NOESY-HSQC (τ_m_ = 50 ms and 120 ms; aliphatic region in ^13^C, with filter elements set to reject protons coupled to ^13^C or ^15^N in F_1_), ^13^C NOESY-HSQC (τ_m_ = 50 ms and 120 ms; aromatic region in ^13^C, with filter elements set to reject protons coupled to ^13^C or ^15^N in F_1_).

For H_2_O samples of complex containing 1:1 natural abundance VSP29 and ^15^N,^13^C-labelled VARP 692–746, the following datasets were acquired: 2D datasets: [^15^N-^1^H] HSQC at 25 °C, 10 °C and 2 °C, [^13^C-^1^H] HSQC (full spectral width in ^13^C); 3D datasets: CBCA(CO)NH, HNCA, HN(CO)CA, HBHA(CO)NH, HNHA, [^1^H-^13^C-^1^H] HCCH-TOCSY, [^13^C-^13^C-^1^H] HCCH-TOCSY, ^15^N NOESY-HSQC (τ_m_ = 150 ms and 70 ms at 25 °C, τ_m_ = 50 ms at 10 °C and 2 °C), ^13^C NOESY-HSQC (τ_m_ = 70 ms; aliphatic region in ^13^C). For ^2^H_2_O samples of complex containing 1:1 natural abundance VSP29 and ^15^N,^13^C-labelled VARP 692–746, the following datasets were acquired: 2D datasets: [^13^C-^1^H] HSQC (aliphatic region in ^13^C), [^13^C-^1^H] HSQC (aromatic region in ^13^C), [^1^H-^13^C-^1^H] HCCH-COSY, [^13^C-^13^C-^1^H] HCCH-TOCSY, ^13^C NOESY-HSQC (τ_m_ = 50 ms, 70 ms and 120 ms; aliphatic region in ^13^C), ^13^C NOESY-HSQC (τ_m_ = 70 ms; aromatic region in ^13^C).

All of the NOESY datasets used for structure calculations (see below) were acquired using pulse sequences modified to ensure equal RF heating in each case, e.g., for ^13^C experiments, a period of ^15^N decoupling equal in length to the acquisition period was applied at the beginning of the inter-scan delay, and for ^15^N experiments an equivalent period of ^13^C decoupling was similarly applied. All spectra were processed using the program TOPSPIN versions 3.1 and 3.2 (Bruker GmbH, Karlsruhe) and analysed using the program CCPN analysis^64^.

### Shift perturbation analysis

Backbone amide group chemical shift perturbations were calculated for VPS29 and for VARP 692–746 using the assignments described above using the formula Δδ =  √((Δδ(^1^H))^2^ + (Δδ (^15^N)/5.0)^2^).

### Structure calculations

Structural models of the VPS29-VARP complex were generated using a hybrid NMR/X-ray crystallographic approach, using simulated annealing calculations run with the program XPLOR-NIH^65^. The conformation of the majority of the VPS29 component was restrained to a template conformation adapted from a previously published X-ray structure (see below), while the conformation of the VARP peptide and interfacial residues of VPS29 (selected on the basis of preliminary structures) were allowed to evolve under a combination of intermolecular and intra-peptide NOE-derived distance restraints, as well as limited NOE-derived restraints for the interfacial region of VPS29 and J-coupling-derived χ^1^ dihedral angle restraints for the interfacial region of the VARP peptide. The NOE restraints were classified into very strong (0–2.3 Å), strong (0–2.9 Å), medium (0–3.5 Å) and weak (0–5.0 Å) intensity categories. The upper distance bounds used for these categories were calibrated using assigned NOE cross-peaks in the unfiltered NOESY spectra of samples in which the Vps29 component was labelled; specifically, the intensity corresponding to the very strong category (0–2.3 Å) was set using sequential d_αN_ peaks in regions of regular anti-parallel β-sheet, that of the strong category (0–2.9 Å) was set using sequential d_NN_ peaks in regions of regular α-helix, that of the medium category (0–3.5 Å) was set using (i, i + 3) d_αN_ peaks in regions of regular α-helix, and those in the weakest category were set to be consistent with the expected approximate detection limit for NOEs, set as 5 Å. This calibration was extended for use in the various types of filtered spectra, in each case by comparing the corresponding measured intensities for particular specific NOE connections as they appeared in both the filtered and unfiltered spectra, using only signals that could be observed free of overlap in both spectra. As a check that the calibration of the intermolecular NOE-based restraints was consistent with the structure calculations and force field, test calculations were also run in which the upper bounds for just these restraints in the final set were either tightened or loosened slightly; it was found that tightening them caused a significant increase in their violations, whereas loosening had relatively little effect since violations were already very few.

The VPS29 template structure was derived from residues 1–181 of the VPS29 component of the VPS29-VPS35 complex in pdb 2R17^66^, with all seleno-Met residues changed to Met and the 11–12, 40–41 and 91–92 peptide bonds set to the *cis* conformation; this structure was selected since it lacks the protruding conformation of helix 2 (95–108) seen in PDB 1Z2X^27^ that makes a crystal contact to its counterpart in a symmetry-related molecule and which is inconsistent with NOE data from the present study^67^. The Leu40-Cys41 peptide bond was modelled in the *cis* conformation because (i) in the highest resolution structure of VPS29 (pdb 5GTU) this peptide was found to be *cis*^43^, and (ii) modelling the *trans* conformation for Leu40-Cys41 led to persistent, severe outliers in the Ramachadran plot for this region both in pdb 2R17 and in trial calculations for our system. The protein co-ordinates from 2R17 were adapted as follows: Hydrogen atoms were first added according to standard geometries, then all atoms except for the guanidinium protons of all arginines and all atoms within 5 Å of residues 40 and 41 were fixed and the structures then subjected to Powell energy minimization (1000 steps), Langevin dynamics at 1000 K (20,000 steps), increase of the van der Waals force constant and tilting of the NOE potential function asymptote in 2000 step cycles, switching to a square-well NOE function then cooling to 300 K in 2000 step cycles and final Powell minimization (1000 steps); this phase of the calculation allowed adaptation to the *cis* conformation of the 40–41 peptide bond as well as resolving atom definition issues for the arginines. All atoms except backbone amide N, C’ and O were then released and the structure subjected to 200 cycles of Powell minimization, after which all atoms were released and the structures again subjected to Powell minimization (100 steps). Only very small movements of the protein backbone occurred during this minimization; for residues 1–181) the backbone co-ordinate shift (rmsd for N, Cα, C’) was 0.150 Å.

To calculate an ensemble of models for the complex, 50 starting structures were first created by randomizing the backbone ϕ and ψ angles of VARP 687–747 and placing the peptide at a random distance (Gaussian distribution around 150 Å) and orientation relative to a copy of the template structure for VPS29. These starting structures were then subjected to a two-stage simulated annealing protocol to generate an ensemble of conformers consistent with the NOE and dihedral angle restraints, all while applying the Ramachandran database potential of mean force^68^ with a force constant of 1 kcal mol^−1^. Since the XPLOR-NIH calculations employed r^−6^ summation for all groups of equivalent protons and non-stereospecifically assigned prochiral groups, and since no stereo-assignments were made (and the assignment-swapping protocol within XPLOR-NIH for deriving stereo-assignments indirectly during the structure calculation itself was not applied), all constraints involving protons within such groups were converted to group constraints (by using wildcards such as HB*). All lower bounds were set to zero^69^. Stage 1 of the two-stage protocol comprised Powell energy minimization (500 steps), Langevin dynamics at 1000 K (5000 steps), increase of the van der Waals force constant and tilting of the NOE potential function asymptote in 1000 step cycles, switching to a square-well NOE function then cooling to 300 K in 500 step cycles and final Powell minimization (500 steps). The force constants used for both the distance and the dihedral angle restraints were 50 kcal mol^−1^. In stage 2, zinc ions were first defined and placed at the geometric average of the co-ordinates of the four zinc-binding cysteinyl sulfur atoms, and all necessary bond and angle terms added to the force field, following which structures were subjected to Powell energy minimization (500 steps), Langevin dynamics at 1000 K while progressively increasing the torsion angle force constant in 1000 and 2000 step cycles, switching to a square-well NOE function then cooling to 300 K in 250 step cycles and final Powell minimization (1000 steps).

Throughout these calculations, strong NCS constraints were used to maintain the internal structure of the VPS29 component. To achieve this the VPS29 structure was duplicated, the co-ordinates of one copy shifted by 500 Å then rigidly fixed and groups of NCS constraints defined between the fixed and unfixed copies so as to maintain the VPS29 structure in the evolving co-ordinates of the unfixed copy. Once preliminary rounds of calculation had established likely regions where contacts between VPS29 and VARP might occur, the NCS terms for just these regions of VPS29 were restricted to backbone atoms and were applied with a much reduced force constant; the NCS constraints used in the final rounds of calculations are shown in Table 1.

Finally, the remaining atoms of the VPS29 N- and C-terminal tails (residues -9-0 and Ser 182) were added in a separate simulated annealing protocol. Initially all atoms of the full structure were placed at fully randomized positions within a 200 Å cube, then for those residues included in the previous stages of the calculations (i.e. residues 1–181 of VPS29 and all residues of the VARP peptide) the randomized co-ordinates were replaced by the previously calculated values and rigidly fixed in place. The structures were then subjected to 1000 cycles of Powell minimization, followed by 5000 steps of Langevin dynamics at 500 K, increase of the van der Waals force constant in 1000 step cycles, cooling to 300 K in 1000 step cycles and final Powell minimization (1000 steps). This protocol for adding the tails and linker was repeated independently five times for each input structure using a different randomization seed each time, and the structure with the lowest value of E(total) retained.

The 25 models with lowest total energy were accepted to form the final ensemble (Fig. 1c). Ramachandran statistics were calculated using the program PROCHECK-NMR^70^ and are as follows: for VARP 710–721: most favoured 60%, additionally allowed 40%, generously allowed 0.0%, disallowed 0.0%; for VPS29 1–181 (this reflects principally the geometry of 2R17 used as a template): most favoured 89.3%, additionally allowed 10.0%, generously allowed 0.0%, disallowed 0.7%. The program CLUSTERPOSE^71^ was used to calculate the mean rmsd of ensembles to their mean structures, and structures were visualized and mean pairwise rmsd values were calculated using the program PYMOL. Ensembles were superposed using the co-ordinates of their respective average structures; the average structures themselves are not shown.

### Mammalian cell culture, siRNA-mediated gene knock-down microscopy

HeLaM cells or HeLaM cells stably expressing VARP-GFP^19^ cultured under standard conditions in RPMI supplemented with 10% (vol/vol) FBS, 2 mM glutamine, 100 U/mL penicillin, and 100 μg/mL streptomycin. siRNA-mediated gene knock-down was performed as described before^19^. In short, cells were transfected (two transfections separated by 48 h) with 100 nM oligonucleotide using Oligofectamine (Life Technologies), according to the manufacturer’s protocol. On-Target Plus oligonucleotide catalogue numbers (Dharmacon/Thermo Scientific) were: non-targeting (NT) control (D-001810-01) and VPS29-1 (J-009764-09). For VPS29 ‘rescue experiments’ and colocalization studies, cells were seeded on glass coverslips and transfected with siRNA-resistant VPS29-TagRFP constructs 24 h after the second knock-down using FuGENE® 6 Transfection Reagent (Roche), according to the manufacturer’s protocol. 24 h after transfection, cells were fixed or incubated for 2 h in fresh cell culture medium prior to fixation (VPS29 ‘rescue experiments’). Knock-down of VPS29 (with actin as a control) and over-expression of VARP-GFP were assessed by immunoblotting using primary antibodies to VPS29 (1:500 Ab 10160, Abcam), actin (1:3000 A2066, Sigma-Aldrich), VARP (1:500 Ab108216, Abcam) and secondary antibodies to IgG (1:1000 anti-goat Ab96938, Abcam and anti-rabbit #926-32221, LI-COR Biosciences).

For immunofluorescence confocal microscopy, cells were fixed with 4% paraformaldehyde in PBS for 10 min. Cells were then permeabilized by incubation with 0.1% (v/v) Triton X-100 in PBS for 5 min and incubated with blocking solution (5% BSA in PBS) for 30 min. This was followed by incubation with primary antibodies used at 1:250 dilution (VPS35, mAB B-5, Santa Cruz; GLUT1, pAb 15309, Abcam; CD107a/LAMP1, mAb 555798, BD Pharmingen, pAb anti-GFP A11122, Molecular Probes) and then fluorescently conjugated secondary antibodies from ThermoFisher used at 1:1000 dilution (Alexa 488 goat anti-rabbit Ig A11034; Alexa 647 goat anti-mouse Ig A21240). Images were captured using a Zeiss LSM 780 confocal microscope as described elsewhere^19,22^. When indicated, the cytosol was extracted prior to fixation by rinsing cells briefly with PBS (with Ca^2+^ and Mg^2+^), followed by incubation for 30 s in 0.05% saponin in PBS (with Ca^2+^ and Mg^2+^), and then immediately formaldehyde fixed. Colocalization of transiently transfected VPS29-TagRFP constructs with either endogenous VPS35 or VARP-GFP was quantified using Pearson’s Correlation Coefficient, calculated using the Zeiss Zen software. Quantitative colocalization of GLUT1 with LAMP1 was performed essentially as previously described^19^ with cell fields randomly selected based on nuclear stain, transfected cells identified by the presence of TagRFP, and focusing using the LAMP1 signal (two independent experiments with 10 fields per condition and a minimum of 20 cells per condition). Single confocal images of cells were acquired corresponding to 1 Airy unit, and the degree of colocalization in individual cells was measured by Manders’ Colocalization (Overlap) Coefficient using Zeiss Zen software. Data are presented as box and whisker plots, in which the boxes extend from the 25th to 75th percentiles, the middle line indicates median. and whiskers represent min to max with all points shown. *p* values were calculated using a one-way Anova with Dunnett’s test in GraphPad Prism 5 (GraphPad Software Inc. La Jolla, CA, USA), with the assumption that each individual cell is a biological replicate.

For immunofluorescence wide field microscopy, cells were fixed, permeabilized and stained with antibodies as for confocal microscopy, except that when staining for Rab7a, 0.25% (v/v) Triton X-100 was used. Additional primary antibodies used at 1:250 dilution for immunofluorescence wide field microscopy were to VPS26 (Ab23892, Abcam), Rab7a (Ab137029, Abcam) and TBC1D5 (Sc376296, Santa Cruz). Fluorescently conjugated secondary antibodies used at 1:1000 dilution were from ThermoFisher (Alexa 555 goat anti-rabbit Ig A21428; Alexa 647 goat anti-mouse Ig A21235). Cells were imaged using a Zeiss AxioPlan microscope with a X63 PlanAPO objective lens and images captured through a Hamamatsu CCD camera controlled via the manufacturer’s software. The Rab7 antibody used does not discriminate between Rab7 in the GDP- or GTP-state, but even if GDP-bound, Rab7 is unlikely removable from tubule membranes through the action of RabGDI due to steric blocking by the polymerized retromer coat.

For immunoelectron microscopy, cells were fixed with 2% paraformaldehyde in 0.1 M phosphate buffer, pH 7.4, and prepared for immunoEM of Tokuyasu frozen sections^72^. Sequential immunolabelling of frozen sections was performed using 1:100 rabbit anti-GFP (ab6556, Abcam, Cambridge, UK) and 1:100 mouse anti-VPS35 (clone B-5, sc-374372, Santa Cruz Biotechnology, Dallas, Texas, USA) as primary antibodies followed by 1:100 rabbit anti-mouse IgG (ab6709, Abcam, Cambridge, UK) and protein A gold (10 nm and 15 nm: Department of Cell Biology, University of Utrecht, The Netherlands). EM sections were examined with a FEI Tecnai G2 Spirit BioTwin TEM (Eindhoven, The Netherlands).

### Evolutionary analysis

VARP homologues were identified using HMMer^73^. A hidden Markov model was generated using phylogenetically verified holozoan VARP homologues published in Herman et al.^56^, which was used to search predicted proteins from the genomes listed in Supplementary Table 2. All hits were then used as queries in BLASTP searches into the *Homo sapiens* genome^74^. A result was considered to be a positive hit if it retrieved VARP or a clear orthologue with an *E*-value < 0.05. A domain analysis to identify ankyrin repeats was performed using the Conserved Domain Database^75^, in order to provide further evidence of VARP orthology.

VPS29 homologues were identified by BLASTP searches using the human VPS29 sequence as a query (NP_476528) to search the genomes listed in Supplementary Table 2. A result was considered to be a positive hit if it was retrieved with an *E*-value  < 0.05, and in a reciprocal search into the human genome, retrieved the query or a clear orthologue as a top hit, also with an *E*-value < 0.05. TBC1D5 proteins were identified by BLASTP using the human sequence as a query (NP_055559), with the same search parameters described for VPS29. As TBC1D5 is closely related to TBC1D13, the identity of putative TBC1D5 sequences was confirmed by phylogenetics.

Alignments were generated for VARP, VPS29, and TBC1D5 families using MUSCLE^76^ with default parameters. All full alignments used to generate supplementary figures are available upon request.

### Reporting summary

Further information on research design is available in the Nature Research Reporting Summary linked to this article.

## Supplementary information

Supplementary Information

Reporting Summary

## Data Availability

Coordinates have been deposited at PDB with code 6TL0, and NMR ^1^H, ^13^C and ^15^N signal assignments have been deposited at BMRB with codes 34461 (VPS29-VARP 692-746 complex), 50107 (free VPS29) and 50108 (free VARP 692-746). Source data are provided with this paper. Other data are available from the corresponding authors upon reasonable request. Source data are provided with this paper.

## Source data

Source Data
