# Supplementary material for: Mechanism and evolution of the Zn-fingernail required for interaction of VARP with VPS29
Source: Nat Commun. 2020 Oct 6;11:5031. doi: 10.1038/s41467-020-18773-2 (PMC7539009; doi:10.1038/s41467-020-18773-2)
Supplement: Supplementary file 1 — Supplementary Information [file 41467_2020_18773_MOESM1_ESM.pdf]

Mechanism and evolution of the VARP:VPS29 interaction : the Zn-fingernail

Crawley-Snowdon et al

Supplementary figures and figure Legends

a

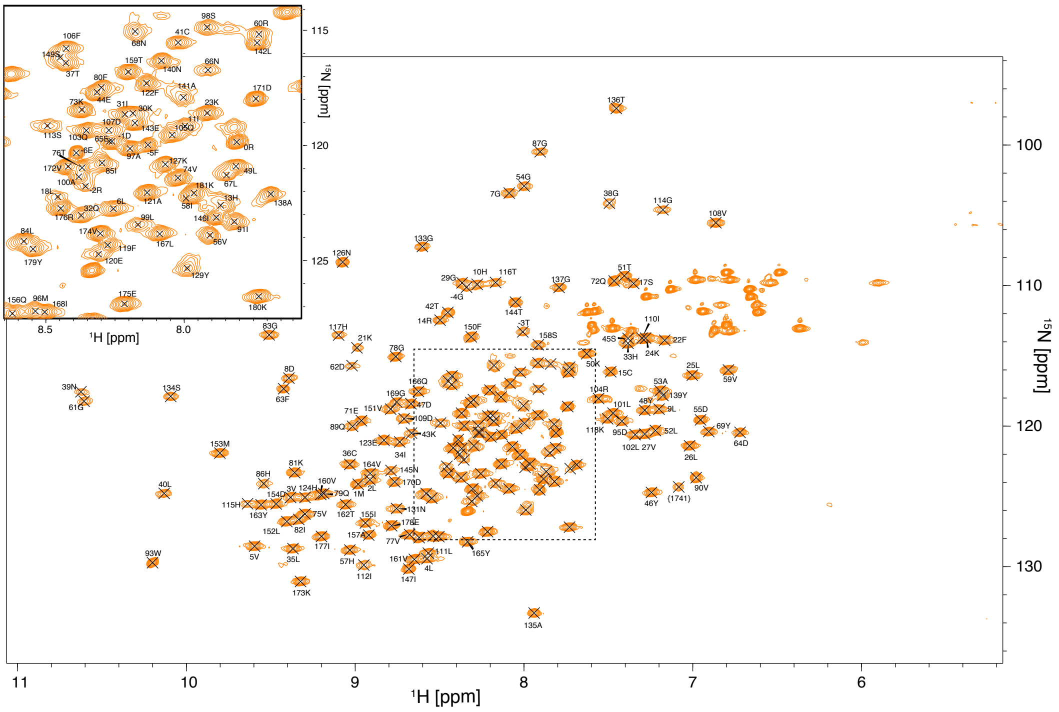

b

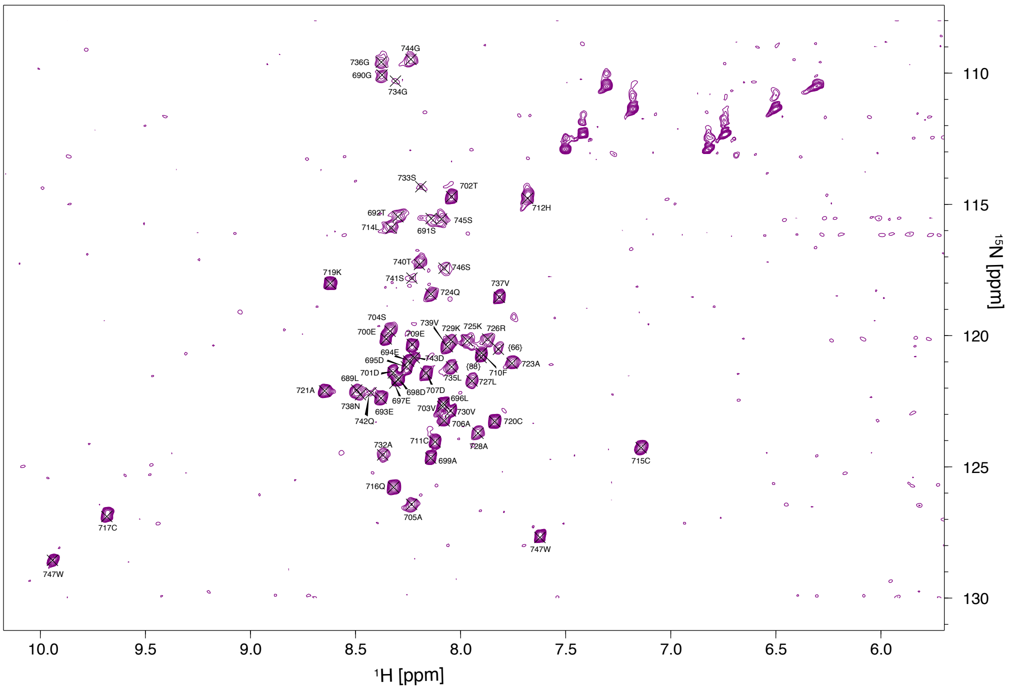

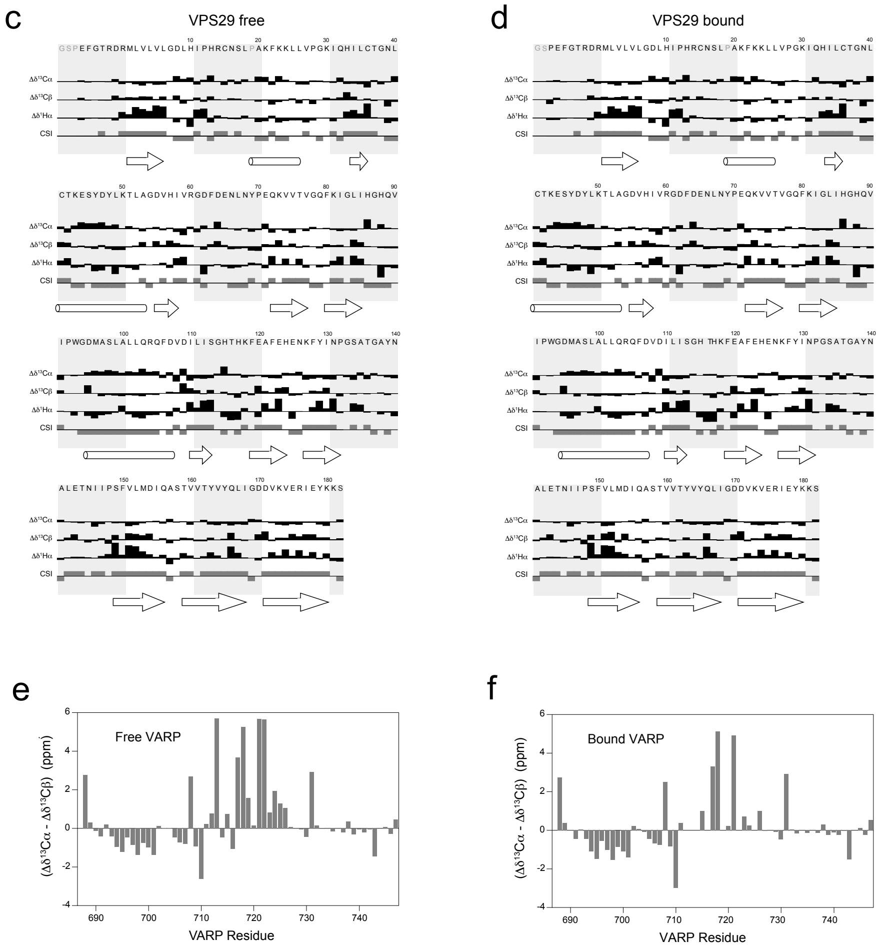

Supplementary Figure 1

Supplementary Figure 1 Zn-fingernail2:VPS29 interaction

a ^{15}N-HSQC spectrum of free ^{15}N-labelled Vps29 in 20mM deuterated Tris, 20mM deuterated DTT, 200mM NaCl, pH7.0, recorded at 800 MHz and 25°C. Backbone amide signal assignments are indicated and the crowded central region is shown as an expansion.

b ^{15}N-HSQC of free ^{15}N-labelled VARP 692-746 in 20mM deuterated Tris, 20mM deuterated DTT, 200mM NaCl, pH7.0, recorded at 800 MHz and 25°C. Backbone amide signal assignments are indicated.

c and d Secondary chemical shift data ($\Delta\delta^{13}C\alpha$, $\Delta\delta^{13}C\beta$ and $\Delta\delta^{1}H\alpha$) for **c** free and **d** bound VPS29. The derived CSI (chemical shift index) data (Wishart and Sykes, 1994) indicate regions of probable α helix (CSI = +1) and β strand (CSI = -1), which correlate well with the actual locations of secondary structural elements in the VPS29 crystal structure (2R17, 5GTU) as shown. Data were calculated using the program CCPN analysis.

e and f Secondary chemical shift data for **e** free and **f** bound VARP residues 692-746 calculated as $\Delta\delta^{13}C\alpha$ - $\Delta\delta^{13}C\beta$ (where $\Delta\delta^{13}C\alpha$ is the difference between the experimentally measured shift of Cα for a given residue and the corresponding random coil value, while $\Delta\delta^{13}C\beta$ is the equivalent quantity for Cβ; values were calculated using the program CCPN analysis). Values substantially above zero indicate the corresponding residue is within the β region of Ramachandran space, whereas values substantially below zero correspondingly indicate α space; values close to zero are consistent with an absence of folded structure.

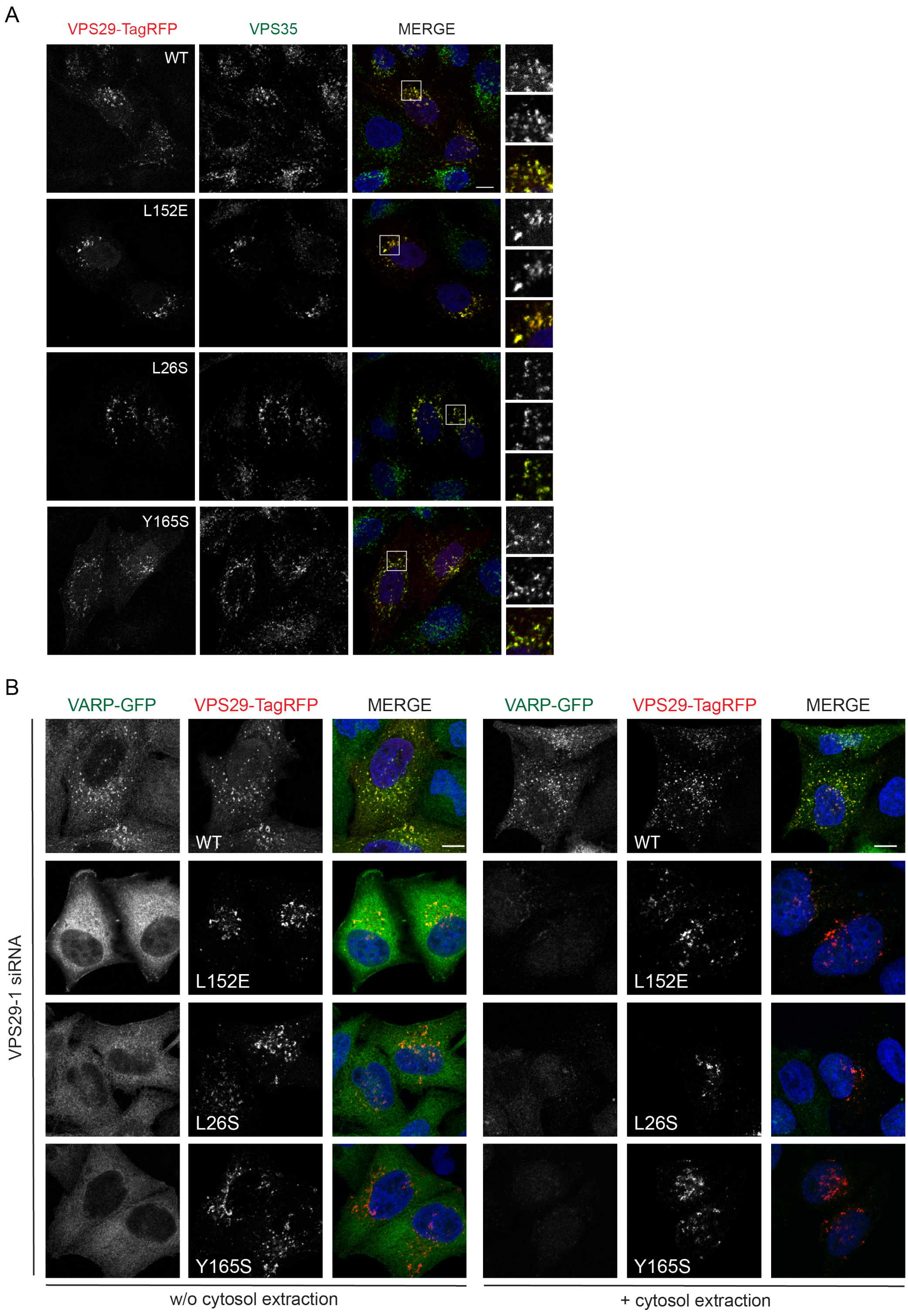

Supplementary Figure 2

Supplementary Figure 2 Colocalization of VPS29-tagRFP with VPS35/VPS26 and TBC1D5.

a HeLaM cells knocked down with VPS29-1 siRNA oligonucleotide and transiently expressing VPS29-tagRFP (wt and mutants L152E, L26S and Y165S) were stained with an antibody to VPS35 and imaged by immunofluorescence confocal microscopy. Representative images are shown with boxed regions from the merged panels expanded and shown as separate VPS29 (top), VPS35 (middle) and merge (bottom) panels on the right. VPS29-TagRFP, red; endogenous VPS35, green, nuclei, blue. Scale bar 10 μm. Mean Pearson's Correlation Coefficients for colocalization of VPS29-tagRFP with endogenous VPS35 were wt, 0.687; L152E, 0.787; L26S, 0.790; Y165S, 0.737.

b HeLaM cells stably expressing VARP-GFP knocked down with VPS29-1 siRNA oligonucleotide and transiently expressing VPS29-TagRFP (wt and mutants L152E, L26S and Y165S), without or with cytosol extraction, were imaged by fluorescence confocal microscopy. Representative images are shown. VARP-GFP, green; transiently transfected VPS29-tagRFP, red; nuclei, blue. Scale bar, 10 μm. In the cytosol extracted cells, Pearson's Correlation Coefficient for colocalization of wt VPS29-tagRFP with VARP-GFP was 0.820 and in cells treated with a non-targeting oligonucleotide as a control 0.753 (images not shown).

a

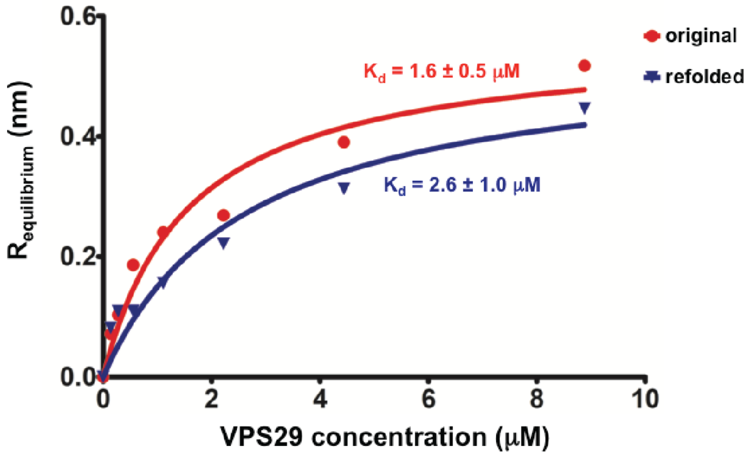

b

VPS29 isoform	Ligand	K_D
Short	VARP 692-746	2.7 μM ± 0.2 μM
Long	VARP 692-746	1.3 μM ± 0.1μM
Short	TBC1D5 132-156	n.d.b.
Long	TBC1D5 132-156	67.3 μM ±8.2 μM

c

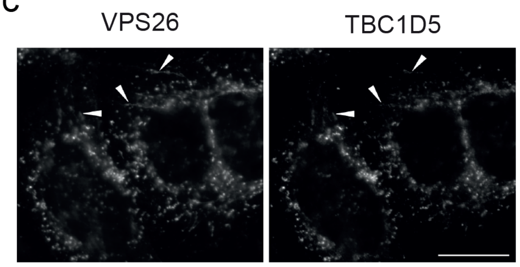

d

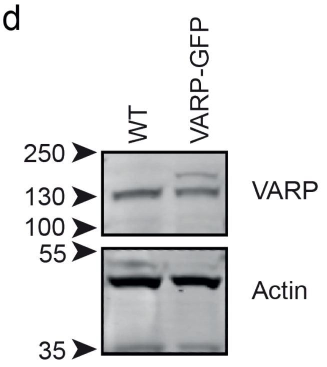

Supplementary Figure 3

Supplementary Figure 3 Retromer TBC1D5 interaction

a Equilibrium analyses by bio-layer interferometry (BIL) and resulting K_Ds of short VPS29 binding to GST-VARP-692-746 immobilized on the biosensor tip, before EDTA treatment (original) and subsequently after treatment with 10mM EDTA and then extensive buffer exchange to re-introduce Zn++ ions (refolded) : resulting KDs are similar 1.6μM vs 2.6 μM.

b K_Ds for the interactions between short and long isoforms of VPS29 with VARP and TBC1D5 ligands as determined by the SPR-based assay described in this study.

c Immunofluorescence wide field microscopy images of HeLaM cells showing that TBC1D5 is found on VPS26-positive endosomes and some of their tubular projections (arrowheads) . Scale bar 20 μm.

d Immunoblots of the HeLaM cells stably expressing VARP-GFP used for the images shown in Figure 4. Blotting with anti-VARP (ab 108216 from Abcam), identified the expected doublet in the stable cells, with the upper GFP-tagged band being 22.4% of the intensity of the lower band, thus indicating overall 1.22-fold over-expression of VARP. Anti-actin (A2066, Signa-Aldrich) was used as a loading control.

```
Scerevisiae       -----------PLMVYVKYKRLSN------------------------------------
Ylipolytica       --------QSSLTCLLDHFGYTQE-----------------------DILADRSVQG---
Mverticillata1    --------LGQPNNEGDTALIIAT--KQIERTTVHGSMASLLGQRMKKQDFDRQNNS---
Pblakesleeanus    --------SSSNLSIARSCLLLSN-----------------------TIYDARDHKG---
Falba             --------QTHTLVFLSLTIRLDSQEAYLVRC-------FQRLQALPLAFFLRPDLGPGS
Cperkinsii        NSEFDECFMCHPLCNCAKCGAISD--KRHSFG---GFKSFNAKFSLHSSVYARNEKG---
Srosetta          --------GCHPLCTCYRCKPVSD------------------------VALLRDAAD---
Cowczarzaki1      --------LCHPLCDCAKCQATLATVGRPHDAPDAPVDDDPIDLDSSITVYSRDGEG---
Cintestinalis     --------KCHPLCECNNCTKLSN--RKANRG-------------SYVSVSSRDDRG---
Hvulgaris         --------MCHPLCSCDSCEKMIL--NMRRDP-------------AAVTVFSRDALG---
Aqueenslandica    --------LCHPLCHCEKCAPIVS--NYHDDP-------------ARVSIYSRDSLK---
Spurpuratus1      --------MCHPLCSCDSCEQILA--SKRNDP-------------RAVSASSRDNRG---
Lchalumnae        --------MCHPLCSCDSCDKLMS--GRLNDP-------------SVVTPYSRDDRG---
Hsapiens          --------MCHPLCFCDDCEKLVS--GRLNDP-------------SVVTPFSRDDRG---
Cmilii            --------MCHPLCFCDNCEKLVS--GRMNDP-------------SIVTPFSRDDRG---
Drerio            --------LCHPLCSCDACDLRIS--GRLNDP-------------SIVTPFSRDDRG---
Ttrahens          ------------------------------------------------------------
Tadhaerens        ------------------------------------------------------------
Lgigantea         ------------------------------------------------------------
Spurpuratus2      ---------ASKFCYGTG------------------------------------------
Cowczarzaki2      ---------SGSPASGRRADLAGSSARGPGRS---------------LSSLSRANST---
Mverticillata2    SSWSRSPLLGPRFSSGTNSPLASPSVSGSTEVPSLNRRQSHTSQPQISQRHSISSGHGHA
```

```
Scerevisiae       ---------ENPKTMEPEEINM--------------------------------------
Ylipolytica       VFLQKWLAHEQPLSWFP-------------------------------------------
Mverticillata1    QCKQESLVDSIFDNYPPVVDDLEHEEKYFEYLNL--------------------------
Pblakesleeanus    KF-------VLSRCIHDLVKKLGHLM----------------------------------
Falba             PAAKPPAGGCSPEALPGAASPEAVLVDSWPTSDFSDISSADGSPLGGEGLFTELLEEGDE
Cperkinsii        PGAK-----CDAICLTSHDTPLSNCKTSSKL-----------------------------
Srosetta          PYAQDK---CHPLCQCERCSRMIRAP---PGSHIT-------------------------
Cowczarzaki1      APTDAVLLMCHPLCQCDKCSMLQSQPSTV----LD-------------------------
Cintestinalis     GLDDDRS--CDPLCQCYKCQPSQDII-------LH-------------------------
Hvulgaris         GFVDESL--CHPLCQCNNCLTVQKKLVNM-ATVLN-------------------------
Aqueenslandica    ---GATA--CHPLCQCEKCQKLRKGHLVF----LN-------------------------
Spurpuratus1      IPLDSKL--CHPLCQCSKCAMLQKR-TTVSASGLS-------------------------
Lchalumnae        YVLKSEF--CHPLCQCPKCEPMQKKLARIPANGLG-------------------------
Hsapiens          SAADPEF--CHPLCQCPKCAPAQKRLAKVPASGLG-------------------------
Cmilii            --LKSEF--CHPLCQCPKCEPAQKKLSSVTVNGLS-------------------------
Drerio            ------------------------------------------------------------
Ttrahens          ------------------------------------------------------------
Tadhaerens        ------------------------------------------------------------
Lgigantea         ------------------------------------------------------------
Spurpuratus2      ------------------------------------------------------------
Cowczarzaki2      ------------------------------------------------------------
Mverticillata2    DKSANSSGNSSPVVEQKEESPKTPSMETPGPKR---------------------------
```

Supplementary Figure 4A

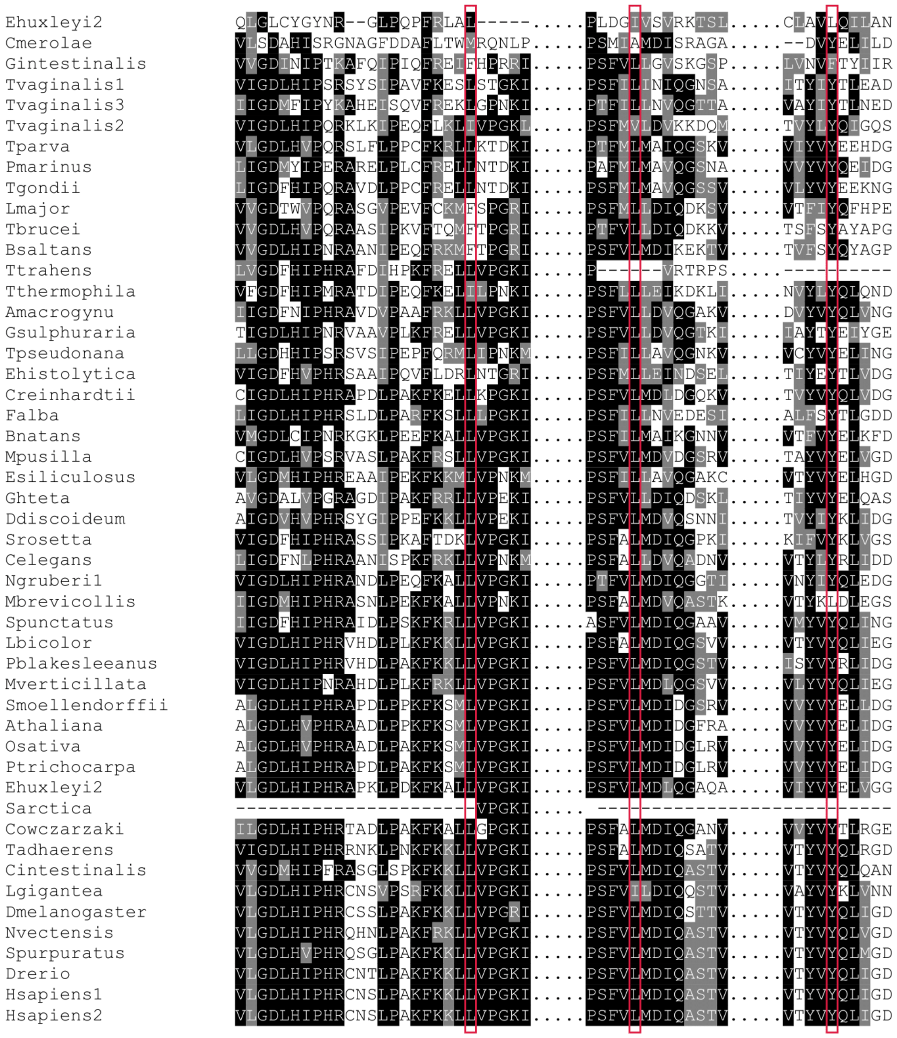

Supplementary Figure 4B

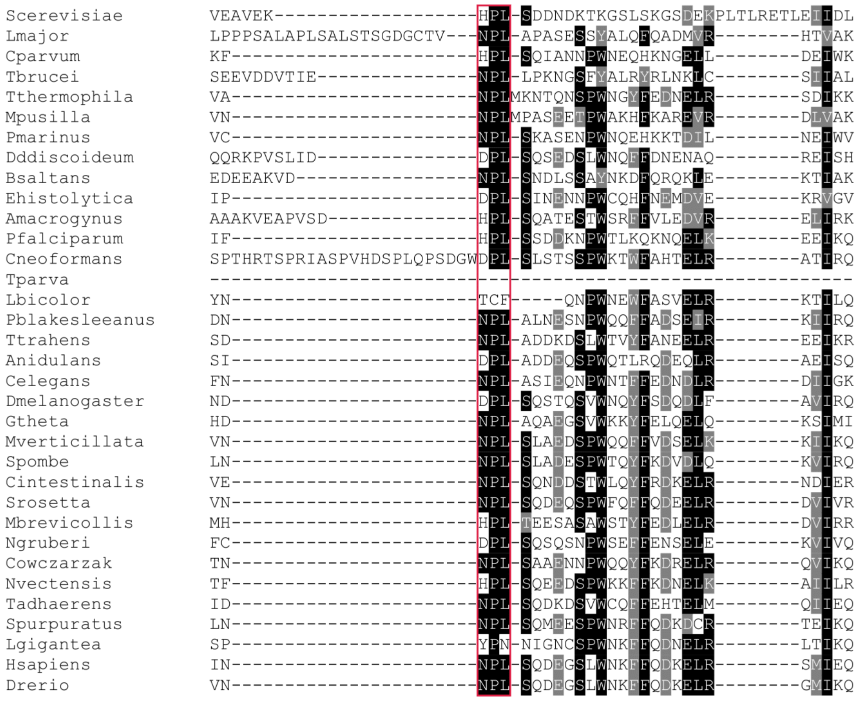

Supplementray Figure 4C

Supplementary Figure 4 Alignments of functionally critical residues in VARP, VPS29 and TBC1D5.

All following position numbers are based on the *H. sapiens* numbering.

a Alignment of VARP homologues from a selection of Opisthokonta taxa. Regions surrounding Zn-fingernail1 (top-positions 2180-2239 of alignment) and Zn-fingernail 2 (bottom-positions 2780-2839 of alignment) are shown.

b Alignment of VPS29 homologues from a diversity of eukaryotes. Leu26, Leu152, and Tyr165 are indicated by red boxes, with flanking sequence. For visualization purposes, intervening sequence between these residues has been removed, as indicated by dots. Top panel= 395-421 of the alignment, middle panel = 735-747 of the alignment, bottom panel = 784-793 of the alignment. The three residues are highly conserved across eukaryotes.

c Alignment of TBC1D5 homologues from a diversity of eukaryotes corresponding to positions 481-540 of the alignment file. The AsnProLeu motif and flanking regions are boxed in red. Although highly conserved, there are occasional changes to a AspProLeuor HisProLeu motif instead.

Supplementary Table 1

Construct	Residues	Cloned
pGEXVARP Zn-fingernail1 wt	396-460 (Human)	BamHI/NotI into pGEX6P1
pGEXVARP Zn-fingernail2 wt and mutants thereof	396-460 (Human)	BamHI/NotI into pGEX6P1
pGEX short VPS29 and mutants thereof	1-182 (mouse)	EcoRI into pGEX4T1
pGEX long VPS29	-4-182 (Mouse)	EcoRI/NotI into pGEX4T1
pGEXTBC1D5	132-156 (Human)	EcoRI/NotI into pGEX4T1
pGEX CLA from β2-adaptin	623 - 632 (Human)	EcoRI/NotI into pGEX4T1
pTagRFP- VPS29 and mutants thereof	1 – 182 (Mouse)	XhoI/BamHI into pTagRFP
pVARP-EGFP	1-1050 (Human)	XhoI/NotI into pLXIN

pGEXVARP Zn-fingernail1, pGEXVARP Zn-fingernail2, pGEX short VPS29, pTagRFP- VPS29, pGEX CLA (residues 623-632 β2-adaptin) were all previously described [19, 22, 77]

pGEX long VPS29, pGEXTBC1D5 (residues 132-156), pGEXshortVPS29 mutants L26S and Y165S, pTagRFP-VPS29 (1-182) mutants L26S and Y165S were cloned using geneblocks (Integrated DNA technologies)

pGEXTBC1D5 (residues 132-156) was separated from GST with a synthetic linker GSAGSASNPNSAGSA encoded in the relevant geneblock in order to overcome problems of lack of accessibility

pVARP-EGFP residues 1-1050 cloned XhoI/NotI into pLXIN

Supplementary Table 2.

Full species names, protein accessions, and accessed databases for sequences included in the alignments shown in Supplementary Figure 4

Varp

Organism	Accession	Alignment name	Database
Saccharomyces cerevisiae	NP_013712.1	Scerevisiae	NCBI
Yarrowia lipolytica	XP_504111.1	Ylipolytica	NCBI
Mortierella verticillata	KFH69979.1	Mverticillata1	NCBI
Phycomyces blakesleeanus	XP_018283856.1	Pblakesleeanus	NCBI
Fonticula alba	XP_009498011.1	Falba	NCBI
Chromosphaera perkinsii	Nk52_evm39s240	Cperkinsii	MulticellGenomeLab
Salpingoeca rosetta	XP_004992668.1	Srosetta	NCBI
Capsaspora owczarzaki	XP_004348666.1	Cowczarzaki1	NCBI
Ciona intestinalis	XP_002123303.2	Cintestinalis	NCBI
Hydra vulgaris	XP_012553826.1	Hvulgaris	NCBI
Amphimedon queenslandica	XP_019850681.1	Aqueenslandica	NCBI
Strongylocentrotus purpuratus	XP_011679328.1	Spurpuratus1	NCBI
Latimeria chalumnae	XP_014349481.1	Lchalumnae	NCBI
Homo sapiens	NP_115515.2	Hsapiens	NCBI
Callorhinchus milii	XP_007887566.1	Cmilii	NCBI
Danio rerio	XP_021326424.1	Drerio	NCBI
Thecamonas trahens	XP_013756626.1	Ttrahens	NCBI
Trichoplax adhaerens	XP_002115177.1	Tadhaerens	NCBI
Lottie gigantea	XP_009066921.1	Lgigantea	NCBI
Strongylocentrotus purpuratus	XP_011671205.1	Spurpuratus2	NCBI
Capsaspora owczarzaki	XP_004343894.2	Cowczarzaki2	NCBI
Mortierella verticillata	KFH73782.1	Mverticillata2	NCBI

Vps29

Organism	Accession	Alignment name	Database
Emiliania huxleyi	XP_005780052.1	Ehuxleyi2	NCBI
Cyanidioschyzon merolae	XP_005538626.1	Cmerolae	NCBI
Giardia intestinalis AWB	XP_001708741.1	Gintestinalis	NCBI
Trichomonas vaginalis	XP_001583400.1	Tvaginalis1	NCBI
Trichomonas vaginalis	XP_001317340.1	Tvaginalis3	NCBI
Trichomonas vaginalis	XP_001318722.1	Tvaginalis2	NCBI
Theileria parva	XP_764436.1	Tparva	NCBI
Perkinsus marinus	XP_002764785.1	Pmarinus	NCBI
Toxoplasma gondii	XP_018638204.1	Tgondii	NCBI
Leishmania major	XP_001685421.1	Lmajor	NCBI
Trypanosoma brucei	XP_829485.1	Tbrucei	NCBI
Bodo saltans	CUI15530.1	Bsaltans	NCBI
Thecamonas trahens	XP_013757779.1	Ttrahens	NCBI
Tetrahymena thermophila	XP_001014758.3	Tthermophila	NCBI

Allomyces macrogynus	KNE55544.1	Amacrogynus	NCBI
Galdieria sulphuraria	XP_005707563.1	Gsulphuraria	NCBI
Thalassiosira pseudonana	XP_002294753.1	Tpseudonana	NCBI
Entamoeba histolytica	XP_652937.2	Ehistolytica	NCBI
Chlamydomonas reinhardtii	XP_001690952.1	Creinhardtii	NCBI
Fonticula alba	XP_009493345.1	Falba	NCBI
Bigelowiella natans	142134	Bnatans	JGI
Micromonas pusilla	XP_003059860.1	Mpusilla	NCBI
Ectocarpus siliculosus	CBJ48594.1	Esiliculosus	NCBI
Guillardia theta	XP_005829471.1	Gtheta	NCBI
Dictyostelium discoideum	XP_636520.1	Ddiscoideum	NCBI
Salpingoeca rosetta	XP_004991697.1	Srosetta	NCBI
Caenorhabditis elegans	NP_001022988.1	Celegans	NCBI
Naegleria gruberi	fgeneshNG_pg.scaffold_71000054	Ngruberi1	JGI
Monosiga brevicollis	XP_001750264.1	Mbrevicollis	NCBI
Spizellomyces punctatus	XP_016604474.1	Spunctatus	NCBI
Laccaria bicolor	XP_001889921.1	Lbicolor	NCBI
Phycomyces blakesleeanus	XP_018293210.1	Pblakesleeanus	NCBI
Mortierella verticillata	KFH66031.1	Mverticillata	NCBI
Selaginella moellendorffii	XP_002969882	Smoellendorffii	NCBI
Arabidopsis thaliana	NP_190365.3	Athaliana	NCBI
Oryza sativa	NP_001046499.1	Osativa	NCBI
Populus trichocarpa	ABK95199.1	Ptrichocarpa	NCBI
Emiliania huxleyi	XP_005774101.1	Ehuxleyi1	NCBI
Salpingoeca arctica	XP_014144991.1	Sarctica	NCBI
Capsaspora owczarzaki	XP_004365000.1	Cowczarzaki	NCBI
Trichoplax adhaerens	XP_002115870.1	Tadhaerens	NCBI
Ciona intestinalis	XP_002130329.1	Cintestinalis	NCBI
Lottia gigantea	XP_009051627.1	Lgigantea	NCBI
Drosophila melanogaster	NP_608575.1	Dmelanogaster	NCBI
Nematostella vectensis	XP_001636588.1	Nvectensis	NCBI
Strongylocentrotus purpuratus	XP_796390.1	Spurpuratus	NCBI
Danio rerio	NP_956331.1	Drerio	NCBI
Homo sapiens	NP_476528.1	Hsapiens1	NCBI
Homo sapiens	NP_057310.1	Hsapiens2	NCBI

TBC1D5

Saccharomyces cerevisiae	NP_012491.3	Scerevisiae	NCBI
Leishmania major	XP_001682404	Lmajor	NCBI
Cryptosporidium parvum	XP_626857	Cparvum	NCBI
Trypanosoma brucei	XP_844946	Tbrucei	NCBI
Tetrahymena thermophila	XP_001032434	Tthermophila	NCBI
Micromonas pusilla	XP_003055805	Mpusilla	NCBI
Perkinsus marinus	XP_002768309	Pmarinus	NCBI
Dictyostelium discoideum	XP_641332	Ddiscoideum	NCBI
Bodo saltans	CUG01591	Bsaltans	NCBI
Entamoeba histolytica	XP_656007	Ehistolytica	NCBI
Allomyces macrogynus	KNE61251	Amacrogynus	NCBI
Plasmodium falciparum	XP_001348873	Pfalciparum	NCBI
Cryptococcus neoformans	XP_024513728	Cneoformans	NCBI
Thalassiosira pseudonana	XP_002295126	Tpseudonana	NCBI
Laccaria bicolor	XP_001875372	Lbicolor	NCBI
Phycomyces blakesleeanus	XP_018290083	Pblakesleeanus	NCBI
Thecamonas trahens	XP_013755543	Ttrahens	NCBI
Aspergillus nidulans	XP_662141	Anidulans	NCBI
Caenorhabditis elegans	NP_497979	Celegans	NCBI
Drosophila melanogaster	NP_731780	Dmelanogaster	NCBI
Guillardia theta	XP_005840216	Gtheta	NCBI
Mortierella verticillata	KFH71770	Mverticillata	NCBI
Schizosaccharomyces pombe	NP_594819	Spombe	NCBI
Ciona intestinalis	XP_009861344	Cintestinalis	NCBI
Salpingoeca rosetta	XP_004992867	Srosetta	NCBI
Monosiga brevicollis	XP_001745400	Mbrevicollis	NCBI
Naegleria gruberi	XP_002680297	Ngruberi	NCBI
Capsaspora owczarzaki	XP_004346254	Cowczarzaki	NCBI
Nematostella vectensis	XP_001634292.1	Nvectensis	NCBI
Trichoplax adhaerens	XP_002109482	Tadhaerens	NCBI
Strongylocentrotus purpuratus	XP_799336	Spurpuratus	NCBI
Lottia gigantea	XP_009059141	Lgigantea	NCBI
Homo sapiens	NP_055559	Hsapiens	NCBI
Danio rerio	NP_001315187	Drerio	NCBI
