# Supplementary material for: Mechanism and evolution of the Zn-fingernail required for interaction of VARP with VPS29
Source: Nat Commun. 2020 Oct 6;11:5031. doi: 10.1038/s41467-020-18773-2 (PMC7539009; doi:10.1038/s41467-020-18773-2)
Supplement: Supplementary file 2 — Reporting Summary [file 41467_2020_18773_MOESM2_ESM.pdf]

nature research

Corresponding author(s): Owen DJ, Neuhaus D, Dacks JB, Luzio JP
NCOMMS-20-03773A

Last updated by author(s): 2020/08/25

Reporting Summary

Nature Research wishes to improve the reproducibility of the work that we publish. This form provides structure for consistency and transparency in reporting. For further information on Nature Research policies, see our Editorial Policies and the Editorial Policy Checklist.

Statistics

For all statistical analyses, confirm that the following items are present in the figure legend, table legend, main text, or Methods section.

n/a	Confirmed	
☐	☒	The exact sample size (*n*) for each experimental group/condition, given as a discrete number and unit of measurement
☐	☒	A statement on whether measurements were taken from distinct samples or whether the same sample was measured repeatedly
☐	☒	The statistical test(s) used AND whether they are one- or two-sided<br>*Only common tests should be described solely by name; describe more complex techniques in the Methods section.*
☒	☐	A description of all covariates tested
☒	☐	A description of any assumptions or corrections, such as tests of normality and adjustment for multiple comparisons
☐	☒	A full description of the statistical parameters including central tendency (e.g. means) or other basic estimates (e.g. regression coefficient) AND variation (e.g. standard deviation) or associated estimates of uncertainty (e.g. confidence intervals)
☐	☒	For null hypothesis testing, the test statistic (e.g. *F*, *t*, *r*) with confidence intervals, effect sizes, degrees of freedom and *P* value noted<br>*Give P values as exact values whenever suitable.*
☒	☐	For Bayesian analysis, information on the choice of priors and Markov chain Monte Carlo settings
☒	☐	For hierarchical and complex designs, identification of the appropriate level for tests and full reporting of outcomes
☒	☐	Estimates of effect sizes (e.g. Cohen's *d*, Pearson's *r*), indicating how they were calculated

Our web collection on statistics for biologists contains articles on many of the points above.

Software and code

Policy information about availability of computer code

Data collection	For NMR, data acquisition on the spectrometer was controlled by the program TopSpin version 3.2 from Bruker BioSpin.
Data analysis	For NMR, off-line data processing used TopSpin version 3.5 from Bruker BioSpin, chemical shift assignment used CcpNmr Analysis version 2.4.2 from CCPN, and structure calculation used X-PLOR NIH version 2.28, from authors Schweiters, Kuszewski, Tjandra and Clore.

For manuscripts utilizing custom algorithms or software that are central to the research but not yet described in published literature, software must be made available to editors and reviewers. We strongly encourage code deposition in a community repository (e.g. GitHub). See the Nature Research guidelines for submitting code & software for further information.

Data

Policy information about availability of data

All manuscripts must include a data availability statement. This statement should provide the following information, where applicable:
- Accession codes, unique identifiers, or web links for publicly available datasets
- A list of figures that have associated raw data
- A description of any restrictions on data availability

6TL0 (co-ordinates for VPS29:VARP 692-746 complex deposited at PDB);
34461 (1H, 13C and 15N NMR signal assignments for VPS29:VARP 692-746 complex deposited at BMRB);
50107 (1H, 13C and 15N NMR signal assignments for free VPS29 deposited at BMRB);
50108 (1H, 13C and 15N NMR signal assignments for free VARP 692-746 deposited at BMRB)

Field-specific reporting

Please select the one below that is the best fit for your research. If you are not sure, read the appropriate sections before making your selection.

- [x] Life sciences
- [ ] Behavioural & social sciences
- [ ] Ecological, evolutionary & environmental sciences

For a reference copy of the document with all sections, see nature.com/documents/nr-reporting-summary-flat.pdf

Life sciences study design

All studies must disclose on these points even when the disclosure is negative.

Sample size	Sample size of individual experiments is indicated in the figure legends and/or methods section
Data exclusions	No data were excluded
Replication	For Fig4a data are shown for individual cells from 2 independent experiments with 10 fields per condition and minimum 20 cells per condition as stated in Methods section. Data are presented as box and whisker plots with mid-line indicating median and all data points shown. p values were calculated using a one-way Anova with Dunnett's test, with the assumption that each individual cell is a biological replicate.
Randomization	For Fig4a cell fields were randomly selected based on nuclear stain.
Blinding	No blinding was applied.

Reporting for specific materials, systems and methods

We require information from authors about some types of materials, experimental systems and methods used in many studies. Here, indicate whether each material, system or method listed is relevant to your study. If you are not sure if a list item applies to your research, read the appropriate section before selecting a response.

Materials & experimental systems

n/a	Involved in the study	
[ ]	[x]	Antibodies
[ ]	[x]	Eukaryotic cell lines
[x]	[ ]	Palaeontology and archaeology
[x]	[ ]	Animals and other organisms
[x]	[ ]	Human research participants
[x]	[ ]	Clinical data
[x]	[ ]	Dual use research of concern

Methods

n/a	Involved in the study	
[x]	[ ]	ChIP-seq
[x]	[ ]	Flow cytometry
[x]	[ ]	MRI-based neuroimaging

Antibodies

Antibodies used

Primary antibodies
VPS35, mAB B-5, sc-374372, lot no. #D0115, Santa Cruz
TBC1D5, Sc376296, lot no. #K1918, Santa Cruz
GLUT1, pAb 15309, lot no. GR3248660-2, Abcam
VPS26, Ab23892, lot no. GR3235809-1, Abcam
Rab7a, Ab137029, lot no. GR155792-38, Abcam
Rabbit anti-GFP, Ab6556, lot no. GR199898-1, Abcam
pAb anti-GFP A11122, lot no. 1024102, Molecular Probes
CD107a/LAMP1, mAb 555798, lot no. 8152608, BD Pharmingen
VPS29, Ab 10160, lot no. GR270492-1, Abcam
VARP, Ab 108216, lot no. GR203344-7, Abcam
Actin, A2066, lot no. 095M4765V, Sigma-Aldrich
Secondary antibodies
Alexa 488 goat anti-rabbit Ig A11034, ThermoFisher
Alexa 647 goat anti-mouse Ig A21240, ThermoFisher
Alexa 555 goat anti-rabbit Ig A21428, ThermoFisher
Alexa 647 goat anti-mouse Ig A21235, ThermoFisher
Rabbit anti-mouse IgG ab6709, Abcam
Donkey anti-goat IgG DyLight650, Ab96938, Abcam
Goat anti-rabbit-IRDye 680, # 926-32221, LI-COR Biosciences

Validation	Primary antibody specificity was as validated by commercial suppliers.<br>VPS35, mAB B-5, sc-374372, Santa Cruz<br>Supplier states: A mouse monoclonal antibody raised against amino acids 497-796 mapping at the C-terminus of human VPS35. Recommended for WB, IP, IF, IHC, ELISA.and stated as suitable for use as control antibody for VPS35 depletion with siRNA.<br>TBC1D5, Sc376296, Santa Cruz<br>Supplier states: A mouse monoclonal antibody specific for an epitope mapping between amino acids 771-794 at the C-terminus of human TBC1D5. Recommended for WB, IP, IF, IHC, ELISA and stated as suitable for use as control antibody for TBC1D5 depletion with siRNA.<br>GLUT1, pAb 15309, Abcam<br>Supplier states: A rabbit polyclonal to a synthetic peptide within Human Glucose Transporter GLUT1 aa 450 to the C-terminus (C terminal). The exact sequence is proprietary. Guarantees use in Flow Cyt, IHC, ICC/IF, WB, IHC-P<br>VPS26, Ab23892, Abcam<br>Supplier states: A rabbit polyclonal to a synthetic peptide corresponding to human VPS26 aa 300 to the C-terminus. Guarantees use in WB, IHC, ICC/IF, IP. KO validated.<br>Rab7a, Ab137029, Abcam<br>Supplier states: A rabbit recombinant monoclonal [EPR7589] to RAB7a.<br>Guarantees use in WB, Flow Cyt, IHC, ICC, IF. KO validated.<br>Rabbit anti-GFP, ab6556, Abcam<br>Supplier states: A rabbit polyclonal antibody to recombinant full length GFP, reactive against all variants of Aequorea victoria GFP including EGFP. Guarantees use in IHC, Electron Microscopy, IHC, IP, WB, ICC/IF, Flow Cyt.<br>pAb anti-GFP A11122, Molecular Probes<br>Supplier states: A rabbit polyclonal antibody to Aequoria GFP tested in IF, IHC, WB, ICC.<br>CD107a/LAMP1, mAb 555798, BD Pharmingen<br>Supplier states: A mouse monoclonal anti-human CD107a, clone H4A3 routinely tested by flow cytometric analysis. States application in flow cytometry and fluorescence microscopy (tested during development).<br>VPS29 Ab 10160, Abcam<br>Supplier states: A goat polyclonal antibody to synthetic peptide DDVKVERIEYKKP, corresponding to C terminal amino acids 170-182 of Human VPS29, suitable for WB. We further validated by siRNA-mediated depletion of endogenous VPS29 in HeLaM cells – see Figure 4c.<br>VARP Ab108216, Abcam<br>Supplier states: A rabbit polyclonal antibody to a synthetic peptide corresponding to a region between amino acids 1000-1050 of human VARP (NP_115515.2). Suitable for WB, IP.<br>Actin A2066, Sigma-Aldrich<br>Supplier states: An affinity purified rabbit polyclonal antibody to a C-terminal fragment of actin that localizes actin in many species and recognizes the 42kDa actin band in immunoblotting of human tissue extracts.

Eukaryotic cell lines

Policy information about cell lines

Cell line source(s)	HeLaM cells were originally a gift from Dr P.J. Lehner, University of Cambridge and were as described in Hewittt EW et al. 2002. EMBO J 21: 2418-2429.
Authentication	Authenticity of the HeLaM cells was verified using the Eurofins cell line authentication service.
Mycoplasma contamination	The HeLaM cells used were negative for mycoplasma contamination. They were routinely tested for the presence of mycoplasma contamination using MycoAlertTM Mycoplasma Detection Kit (LT07-318, Lonza), and were treated with mycoplasma removing agent (BM-Cyclin from Roche, cat. no. 10 799 050 001).
Commonly misidentified lines<br>(See ICLAC register)	No commonly misidentified cell lines were used.
